# Supplementary material for: Novel Thiazole Phenoxypyridine Derivatives Protect Maize from Residual Pesticide Injury Caused by PPO-Inhibitor Fomesafen
Source: Biomolecules. 2019 Sep 20;9(10):514. doi: 10.3390/biom9100514 (PMC6843476; doi:10.3390/biom9100514)

checkCIF/PLATON report

Structure factors have been supplied for datablock(s) mo_DBND_1_0ma_a

THIS REPORT IS FOR GUIDANCE ONLY. IF USED AS PART OF A REVIEW PROCEDURE FOR PUBLICATION, IT SHOULD NOT REPLACE THE EXPERTISE OF AN EXPERIENCED CRYSTALLOGRAPHIC REFEREE.

No syntax errors found.   CIF dictionary   Interpreting this report

Datablock: mo_DBND_1_0ma_a

```
Bond precision:  C-C = 0.0062 A          Wavelength=0.71073

Cell:            a=8.0896(3)     b=12.0540(5)     c=42.6668(14)
                 alpha=90        beta=90          gamma=90
Temperature:     100 K
```

	Calculated	Reported
Volume	4160.5(3)	4160.5(3)
Space group	P 21 21 21	P 21 21 21
Hall group	P 2ac 2ab	P 2ac 2ab
Moiety formula	C20 H19 F3 N2 O4 S	C20 H19 F3 N2 O4 S
Sum formula	C20 H19 F3 N2 O4 S	C20 H19 F3 N2 O4 S
Mr	440.43	440.43
Dx,g cm-3	1.406	1.406
Z	8	8
Mu (mm-1)	0.211	0.211
F000	1824.0	1824.0
F000’	1826.11	
h,k,lmax	10,15,55	10,15,55
Nref	9586[5408]	9573
Tmin,Tmax	0.973,0.979	0.973,0.979
Tmin’	0.973	

```
Correction method= # Reported T Limits: Tmin=0.973 Tmax=0.979
AbsCorr = MULTI-SCAN

Data completeness= 1.77/1.00      Theta(max)= 27.510

R(reflections)= 0.0547( 7518)     wR2(reflections)= 0.1636( 9573)

S = 1.117                  Npar= 547
```

The following ALERTS were generated. Each ALERT has the format
test-name_ALERT_alert-type_alert-level.
Click on the hyperlinks for more details of the test.

Alert level C

```
ABSTY02_ALERT_1_C  An _exptl_absorpt_correction_type has been given without
            a literature citation. This should be contained in the
            _exptl_absorpt_process_details field.
            Absorption correction given as multi-scan
PLAT213_ALERT_2_C Atom F2              has ADP max/min Ratio .....        3.6 prolat
PLAT234_ALERT_4_C Large Hirshfeld Difference F6       --C21              0.16 Ang.
PLAT241_ALERT_2_C High   'MainMol' Ueq as Compared to Neighbors of         O5 Check
PLAT242_ALERT_2_C Low    'MainMol' Ueq as Compared to Neighbors of        C19 Check
PLAT340_ALERT_3_C Low Bond Precision on  C-C Bonds ...............    0.00619 Ang.
PLAT910_ALERT_3_C Missing # of FCF Reflection(s) Below Theta(Min).          7 Note
PLAT934_ALERT_3_C Number of (Iobs-Icalc)/SigmaW > 10 Outliers ....          1 Check
```

Alert level G

```
PLAT066_ALERT_1_G Predicted and Reported Tmin&Tmax Range Identical          ? Check
PLAT242_ALERT_2_G Low    'MainMol' Ueq as Compared to Neighbors of         C1 Check
PLAT242_ALERT_2_G Low    'MainMol' Ueq as Compared to Neighbors of        C21 Check
PLAT791_ALERT_4_G Model has Chirality at C14         (Chiral SPGR)          R Verify
PLAT791_ALERT_4_G Model has Chirality at C34         (Chiral SPGR)          R Verify
PLAT912_ALERT_4_G Missing # of FCF Reflections Above STh/L=  0.600          2 Note
PLAT978_ALERT_2_G Number C-C Bonds with Positive Residual Density.          1 Info
```

0 **ALERT level A** = Most likely a serious problem - resolve or explain
0 **ALERT level B** = A potentially serious problem, consider carefully
8 **ALERT level C** = Check. Ensure it is not caused by an omission or oversight
7 **ALERT level G** = General information/check it is not something unexpected

2 ALERT type 1 CIF construction/syntax error, inconsistent or missing data
6 ALERT type 2 Indicator that the structure model may be wrong or deficient
3 ALERT type 3 Indicator that the structure quality may be low
4 ALERT type 4 Improvement, methodology, query or suggestion
0 ALERT type 5 Informative message, check

It is advisable to attempt to resolve as many as possible of the alerts in all categories. Often the minor alerts point to easily fixed oversights, errors and omissions in your CIF or refinement strategy, so attention to these fine details can be worthwhile. In order to resolve some of the more serious problems it may be necessary to carry out additional measurements or structure refinements. However, the purpose of your study may justify the reported deviations and the more serious of these should normally be commented upon in the discussion or experimental section of a paper or in the "special_details" fields of the CIF. checkCIF was carefully designed to identify outliers and unusual parameters, but every test has its limitations and alerts that are not important in a particular case may appear. Conversely, the absence of alerts does not guarantee there are no aspects of the results needing attention. It is up to the individual to critically assess their own results and, if necessary, seek expert advice.

Publication of your CIF in IUCr journals

A basic structural check has been run on your CIF. These basic checks will be run on all CIFs submitted for publication in IUCr journals (*Acta Crystallographica*, *Journal of Applied Crystallography*, *Journal of Synchrotron Radiation*); however, if you intend to submit to *Acta Crystallographica Section C* or *E* or *IUCrData*, you should make sure that full publication checks are run on the final version of your CIF prior to submission.

Publication of your CIF in other journals

Please refer to the *Notes for Authors* of the relevant journal for any special instructions relating to CIF submission.

PLATON version of 23/04/2018; check.def file version of 23/04/2018

Datablock mo_DBND_1_0ma_a - ellipsoid plot

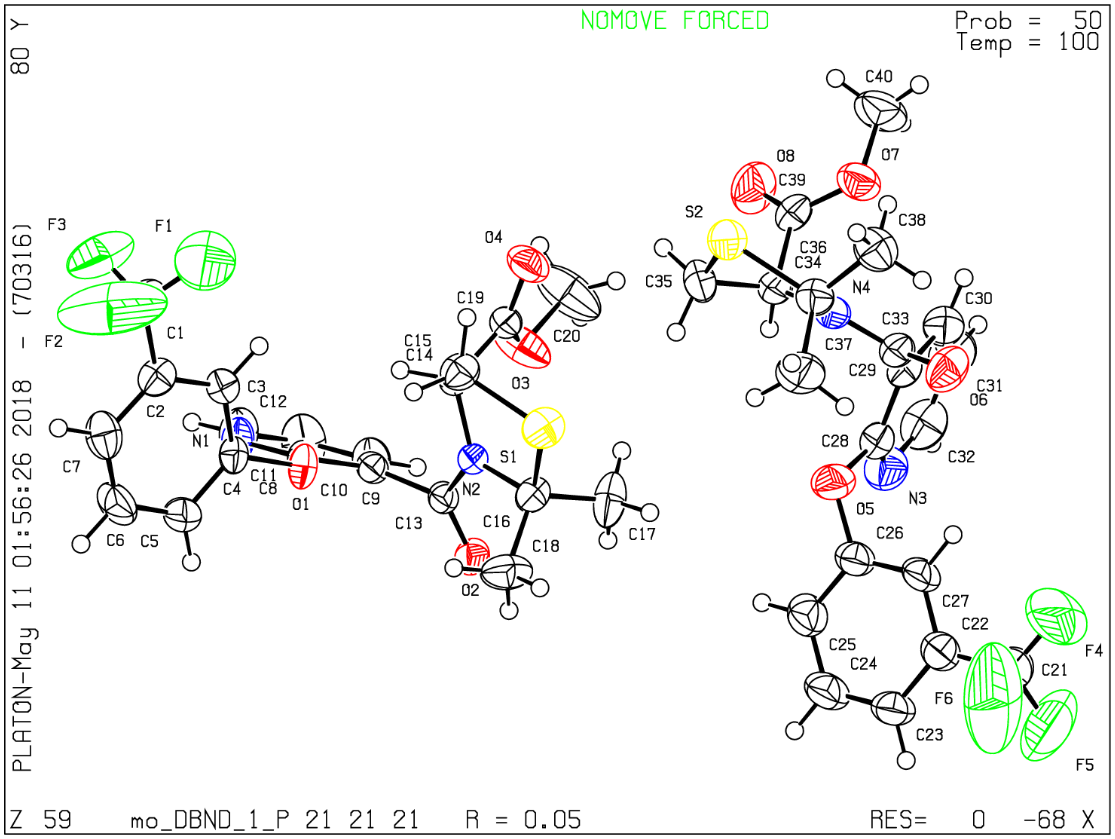

Supplement: Supplementary file 1 [file biomolecules-09-00514-s001.zip › Crystallographic Data for Compound 4a/checkcif.pdf]
