# Supplementary material for: Novel Thiazole Phenoxypyridine Derivatives Protect Maize from Residual Pesticide Injury Caused by PPO-Inhibitor Fomesafen
Source: Biomolecules. 2019 Sep 20;9(10):514. doi: 10.3390/biom9100514 (PMC6843476; doi:10.3390/biom9100514)
Supplement: Supplementary file 1 [file biomolecules-09-00514-s001.zip › Supplementary Material.pdf]

*Supplementary Material*

# Novel Thiazole Phenoxypyridines Derivatives Protect Maize from Residual Pesticide Injury Caused by PPO-inhibitor Fomesafen

**Li-xia Zhao, Min-lei Yin, Qing-rui Wang, Yue-li Zou, Tao Ren, Shuang Gao, Ying Fu * and Fei Ye ***

Department of Applied Chemistry, College of Science, Northeast Agricultural University, Harbin, 150030, China;

* Correspondence: fuying@neau.edu.cn (Y. F.); yefei@neau.edu.cn (F. Y.)

**Supplementary Information**

# Compound 4a

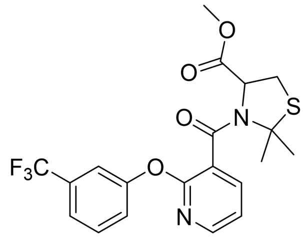

*Methyl(R)-2,2-dimethyl-3-(2-(3-(trifluoromethyl)phenoxy)nicotinoyl)thiazolidine-4-carboxylate*

# IR

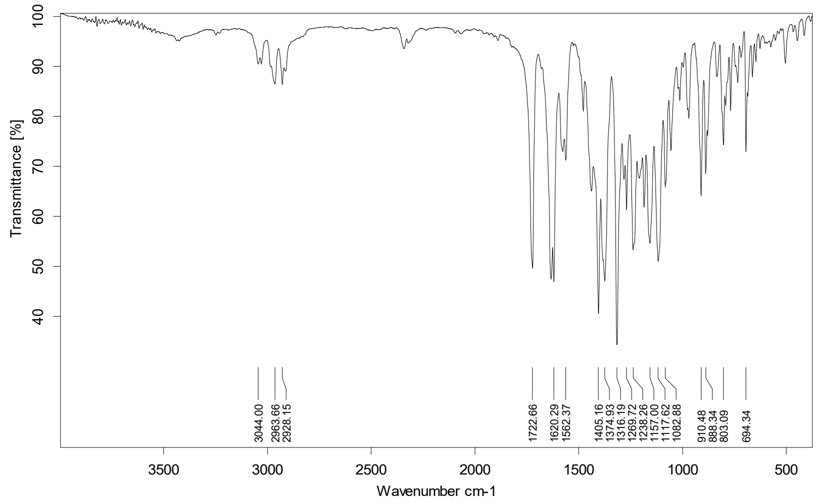


# $^1$H NMR

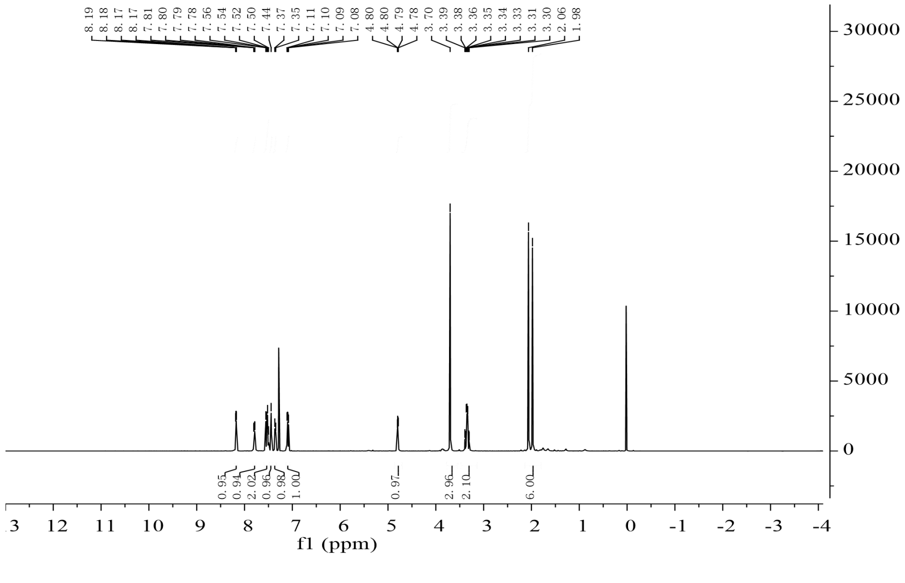

## $^{13}$C NMR

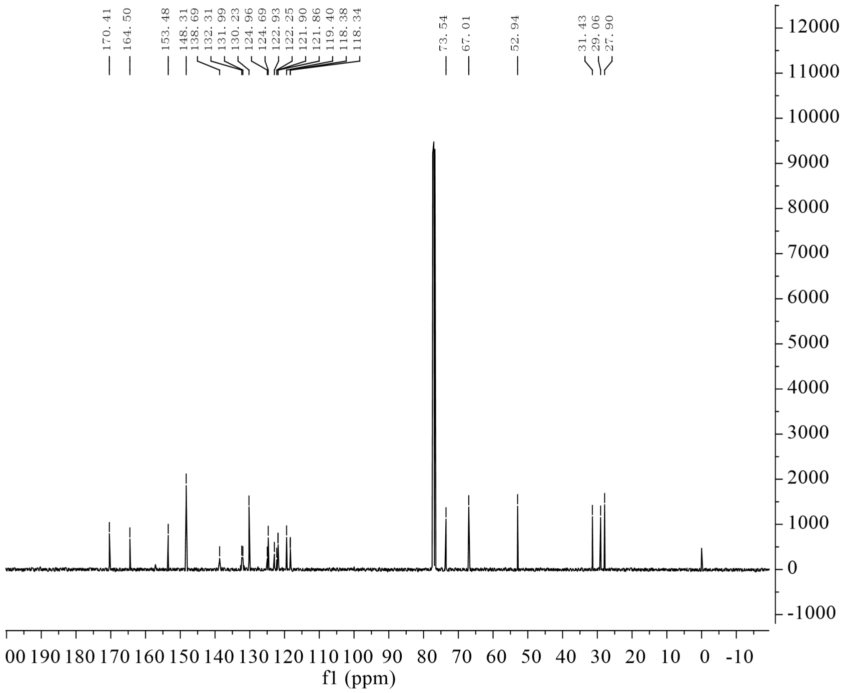

## HRMS

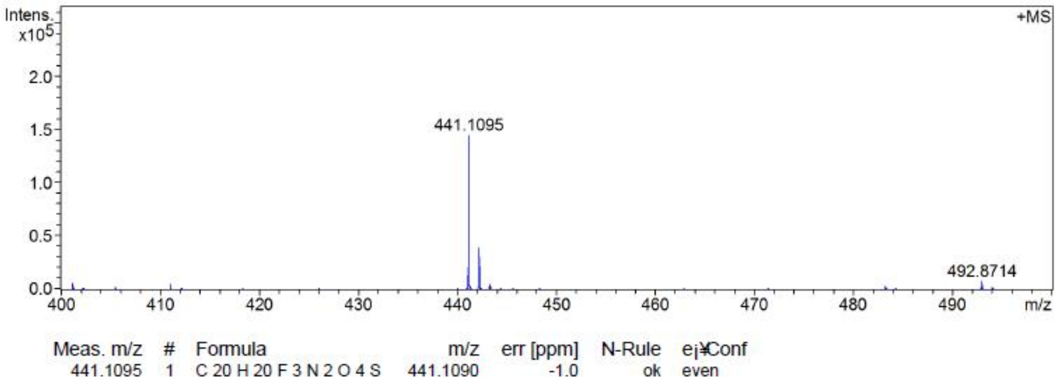

White solid, m.p. 84-85 °C; IR (KBr, cm$^{-1}$) $\nu$: 3044-2828 (C-H), 1722, 1620 (C=O), 1562-1405 (C=C), 1269 (C-O); $^{1}$H NMR (400 MHz, $CDCl_3$) δ 8.18 (dd, *J* = 4.8, 1.9 Hz, 1H, Py-H), 7.80 (dd, *J* = 7.4, 1.6 Hz, 1H, Py-H), 7.53 (dd, *J* = 14.2, 7.8 Hz, 2H, Ar-H), 7.44 (s, 1H, Ar-H), 7.36 (d, *J* = 7.8 Hz, 1H, Ar-H), 7.10 (dd, *J* = 7.4, 5.0 Hz, 1H, Py-H), 4.79 (dd, *J* = 5.4, 2.1 Hz, 1H, N-CH), 3.70 (s, 3H, O-$CH_3$), 3.35 (m, 2H, S-$CH_2$), 2.02 (d, *J* = 33.4 Hz, 6H, C-$(CH_3)_2$); $^{13}$C NMR (101 MHz, $CDCl_3$) δ 170.41, 164.50, 153.48, 148.31, 138.69, 130.23, 124.69, 122.93, 121.90, 121.86, 119.40, 118.38, 118.34, 73.54, 67.01, 52.94, 31.43, 29.06, 27.90; HRMS calcd. for [M + H$^{+}$] $C_{20}H_{20}F_3N_2O_4S$: 441.1090, found 441.1095.

# Compound 4b

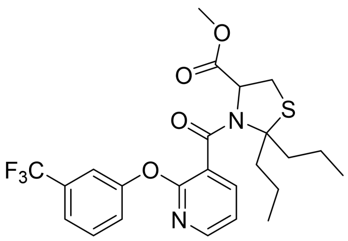


*Methyl (R)-4-(2-(3-(trifluoromethyl)phenoxy)nicotinoyl)-1-thia-4-azaspiro [4.5]decane-3-carboxylate*

# IR

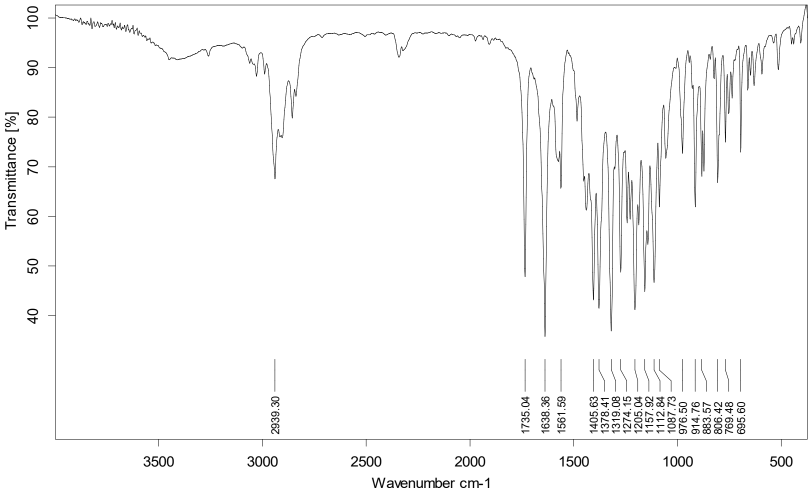


# $^{1}$H NMR

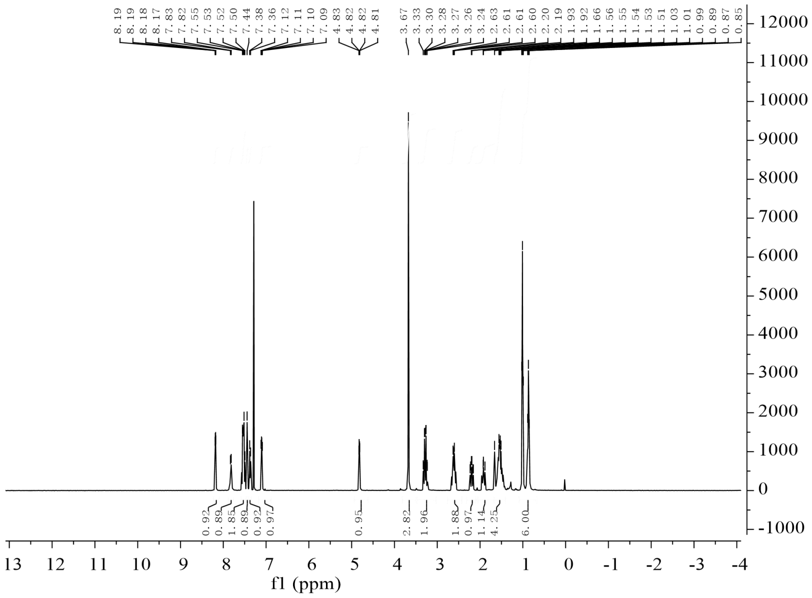

## $^{13}$C NMR

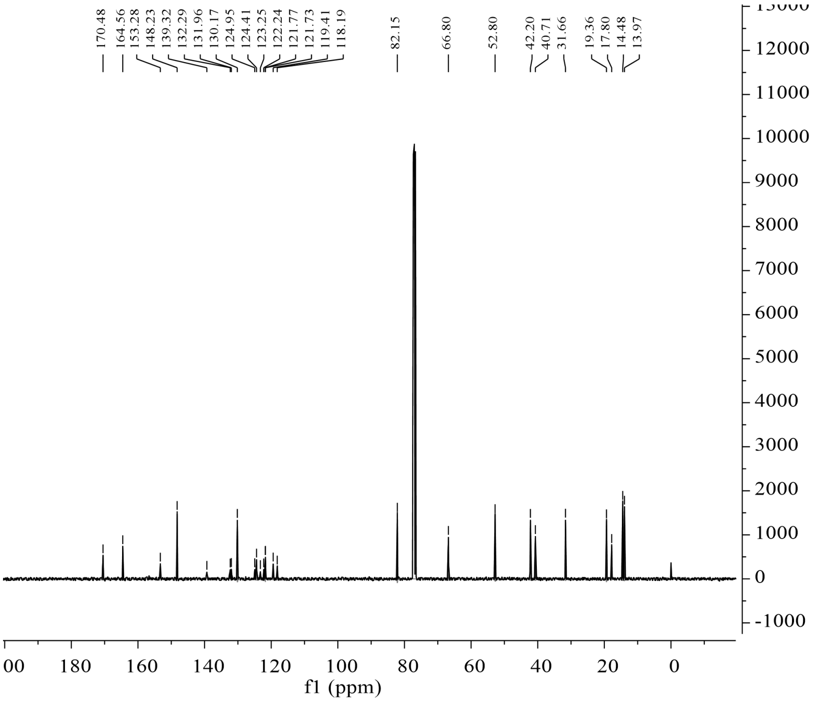


## HRMS

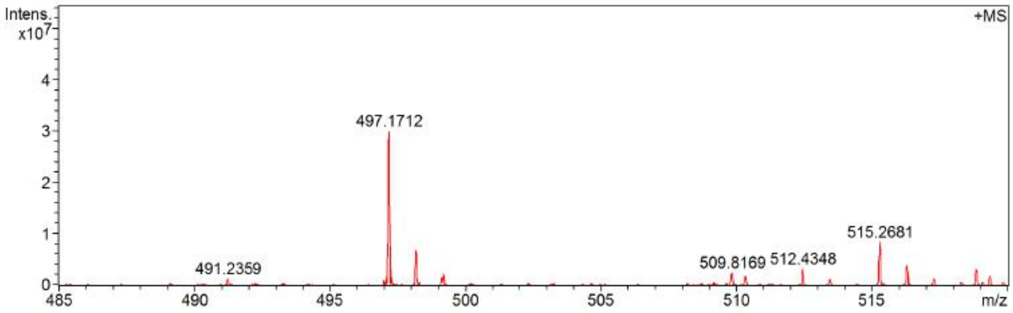


| Meas. m/z | # | Formula | m/z | err [ppm] | N-Rule | e¦¥Conf |
|---|---|---|---|---|---|---|
| 497.1712 | 1 | C 24 H 28 F 3 N 2 O 4 S | 497.1716 | 0.9 | ok | even |

White solid, m.p. 121-122 °C; IR (KBr, cm$^{-1}$) *ν*: 2939 (C-H), 1735, 1638 (C=O), 1561-1405 (C=C), 1274 (C-O); $^{1}$H NMR (400 MHz, $CDCl_3$) δ 8.22-8.13 (m, 1H, Py-H), 7.77 (s, 1H, Py-H), 7.52 (dd, *J* = 13.4, 7.4 Hz, 2H, Ar-H), 7.44 (s, 1H, Ar-H), 7.35 (d, *J* = 7.2 Hz, 1H, Ar-H), 7.10 (t, *J* = 5.2 Hz, 1H, Py-H), 4.82 (s, 1H, N-CH), 3.71 (s, 3H, O-$CH_3$), 3.25-3.02 (m, 2H, S-$CH_2$), 2.61-1.92 (m, 4H, C-$CH_2$-), 1.66-1.51 (m, 4H,-C-$CH_2$-), 0.95-0.99 (m, 6H, -C-$CH_3$); $^{13}$C NMR (101 MHz, $CDCl_3$) δ 170.56, 164.64, 153.53, 148.17, 130.20, 124.66, 121.85, 119.42, 118.32, 67.09, 52.91, 36.02, 34.43, 30.81, 25.48, 25.41, 24.54; HRMS calcd. for [M + H$^{+}$] $C_{24}H_{28}F_3N_2O_4S$: 497.1716, found 497.1716.

# Compound 4c

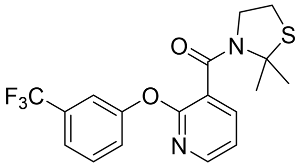


*(2,2-dimethylthiazolidin-3-yl)(2-(3-(trifluoromethyl)phenoxy)pyridin-3-yl)methanone*

# IR

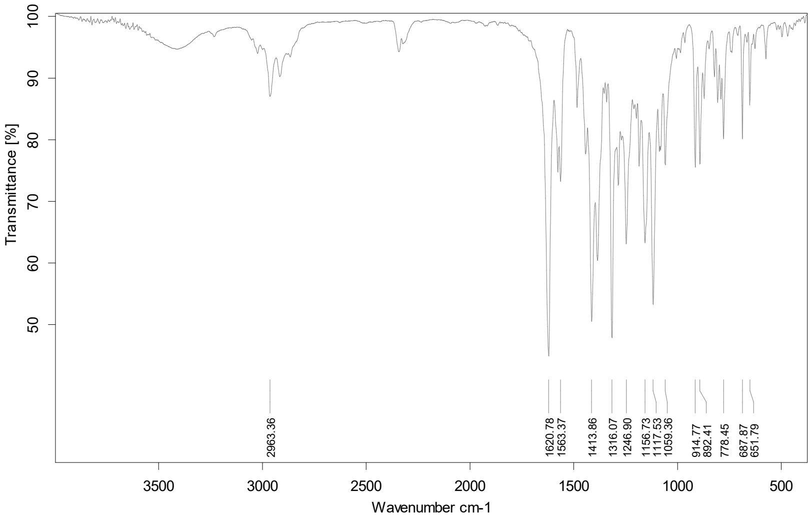


# $^1$H NMR

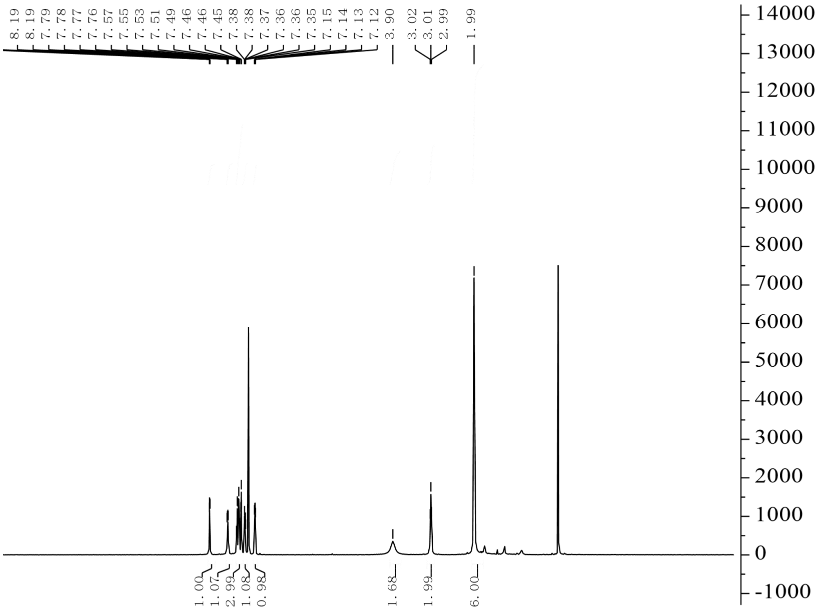

## $^{13}$C NMR

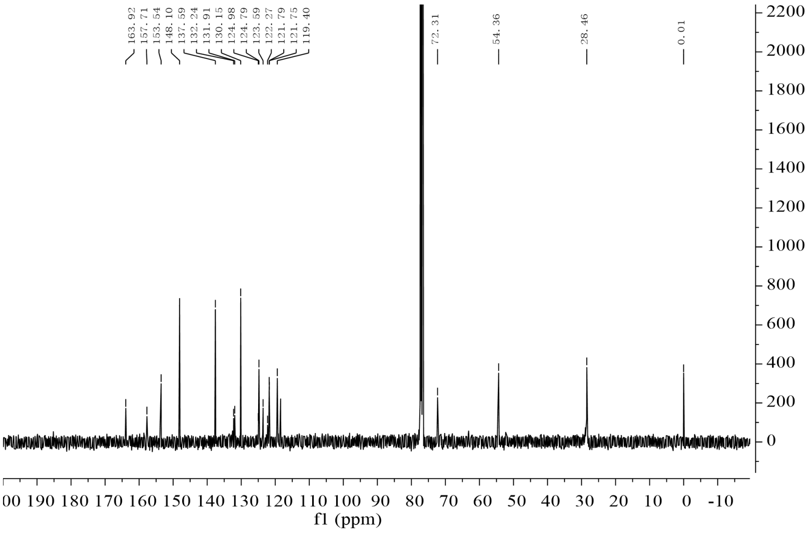


## HRMS

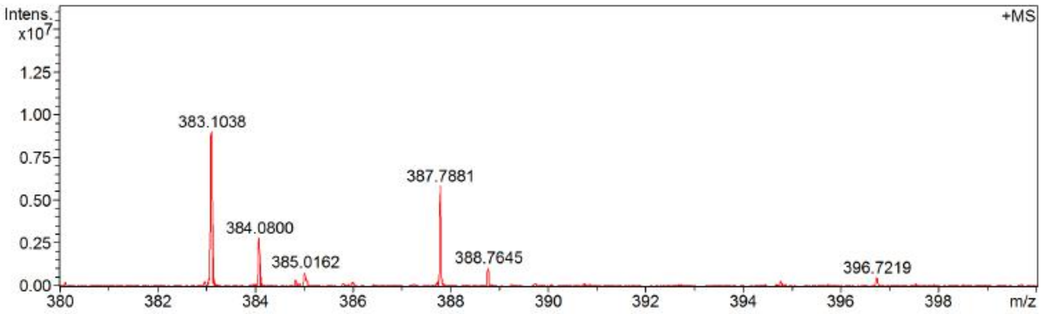


| Meas. m/z | # | Formula | m/z | err [ppm] | N-Rule | e¡¥Conf |
|---|---|---|---|---|---|---|
| 383.1038 | 1 | C 18 H 18 F 3 N 2 O 2 S | 383.1036 | -0.6 | ok | even |

White solid, m.p. 70-71 °C; IR (KBr, cm$^{-1}$) $v$: 2963 (C-H), 1620 (C=O), 1563-1413 (C=C), 1246 (C-O); $^{1}$H NMR (400 MHz, $CDCl_3$) δ 8.20 (dd, $J$ = 5.0, 1.9 Hz, 1H, Py-H), 7.78 (dd, $J$ = 7.4, 1.9 Hz, 1H, Py-H), 7.58-7.42 (m, 3H, Ar-H), 7.37 (dt, $J$ = 7.9, 1.8 Hz, 1H, Ar-H), 7.13 (dd, $J$ = 7.3, 4.9 Hz, 1H, Py-H), 3.90 (s, 2H, N-$CH_2$), 3.01 (t, $J$ = 6.0 Hz, 2H, S-$CH_2$), 1.99 (s, 6H, $C(CH_3)_2$); $^{13}$C NMR (101 MHz, $CDCl_3$) δ 163.92, 157.71, 153.54, 148.10, 137.59, 130.15, 124.79, 123.59, 121.79, 121.75, 119.40, 72.31, 54.36, 28.46; HRMS calcd. for [M + H$^{+}$] $C_{18}H_{18}$ $F_3N_2O_2S$: 383.1036, found 383.1038.

# Compound 4d

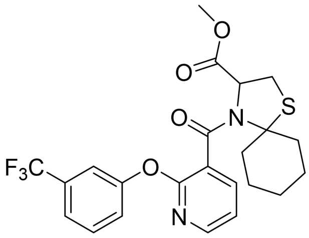

*Methyl (R)-4-(2-(3-(trifluoromethyl)phenoxy)nicotinoyl)-1-thia-4-azaspiro [4.5]decane-3-carboxylate*

# IR

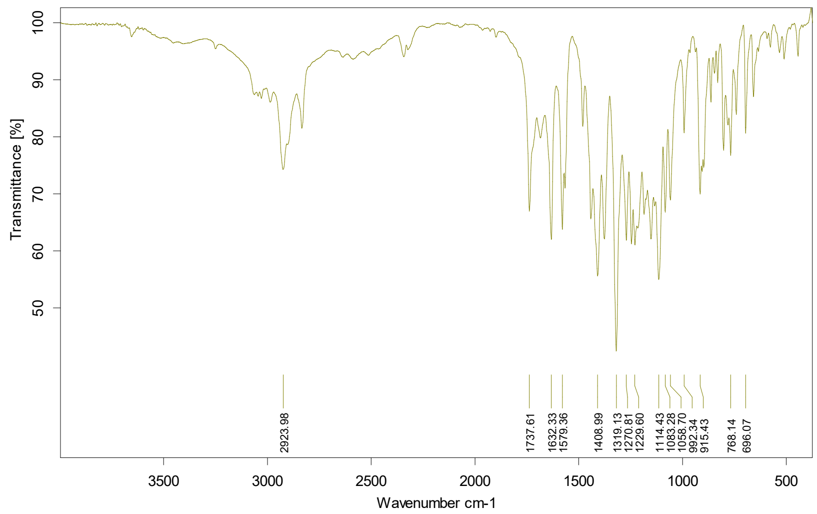


# $^{1}$H NMR

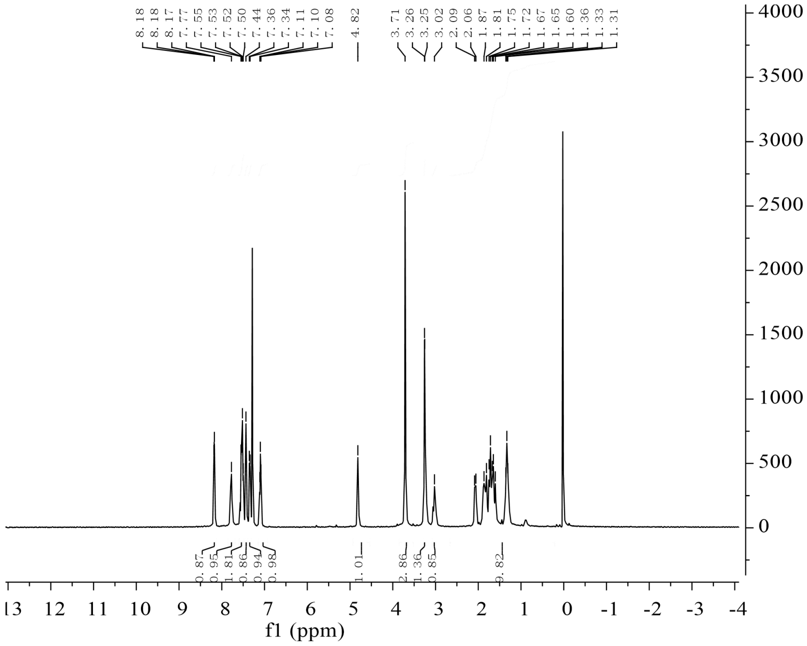


# $^{13}$C NMR

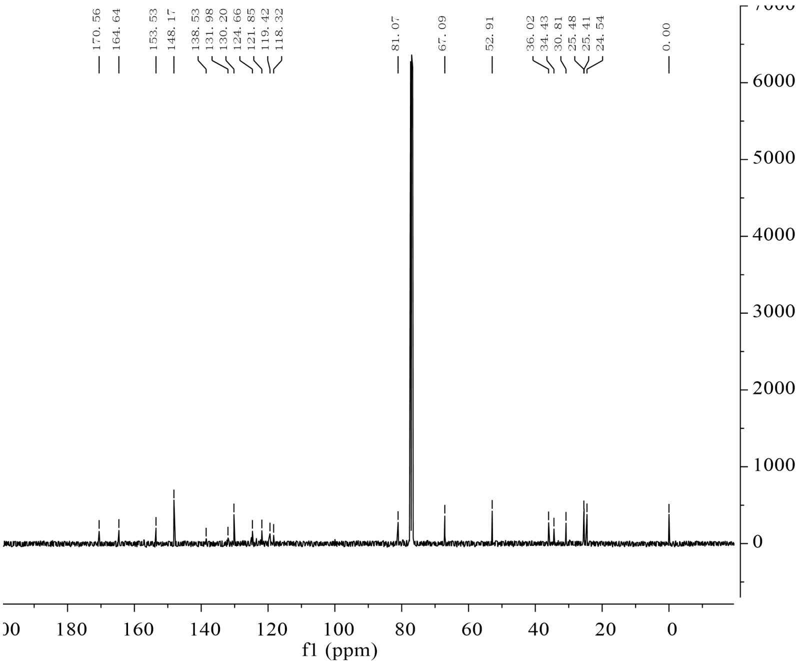


# HRMS

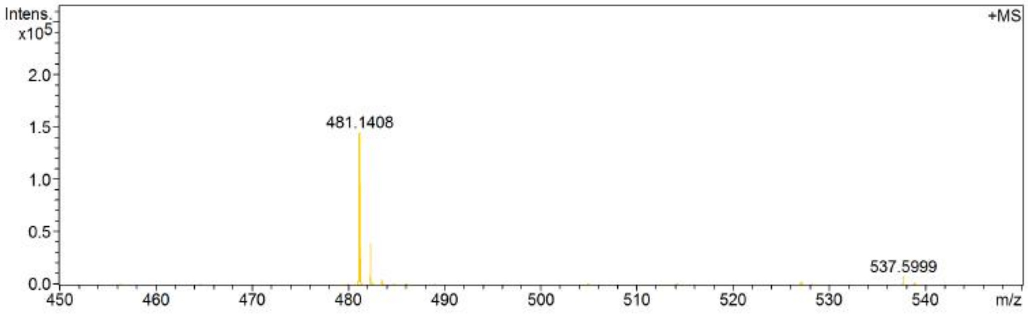


| Meas. m/z | # | Formula | m/z | err [ppm] | N-Rule | e¡¥Conf |
|---|---|---|---|---|---|---|
| 481.1408 | 1 | C 23 H 24 F 3 N 2 O 4 S | 481.1403 | -1.0 | ok | even |

White solid, m.p. 127-128 °C; IR (KBr, cm$^{-1}$) $\nu$:2923 (C-H), 1737,1638 (C=O), 1561-1405 (C=C), 1270 (C-O); $^{1}$H NMR (400 MHz, $CDCl_3$) δ 8.22-8.13 (m, 1H, Py-H), 7.77 (s, 1H, Py-H), 7.52 (dd, *J* = 13.4, 7.4 Hz, 2H, Ar-H), 7.44 (s, 1H, Ar-H), 7.35 (d, *J* = 7.2 Hz, 1H, Ar-H), 7.10 (t, *J* = 5.2 Hz, 1H, Py-H), 4.82 (s, 1H, N-CH), 3.71 (s, 3H, $OCH_3$), 3.25-3.02 (m, 2H, S-$CH_2$), 2.12-1.31 (m, 10 H, -C-$CH_3$); $^{13}$C NMR (101 MHz, $CDCl_3$) δ 170.56, 164.64, 153.53, 148.17, 130.20, 124.66, 121.85, 119.42, 118.32, 67.09, 52.91, 36.02, 34.43, 30.81, 25.48, 25.41, 24.54; HRMS calcd. for [M + H$^{+}$] $C_{23}H_{24}F_3N_2O_4S$: 481.1403, found 481.1408.

# Compound 4e

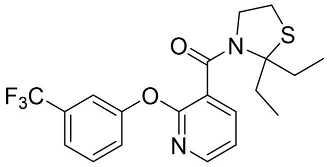

*(2,2-diethylthiazolidin-3-yl)(2-(3-(trifluoromethyl)phenoxy)pyridin-3-yl)methanone*

# IR

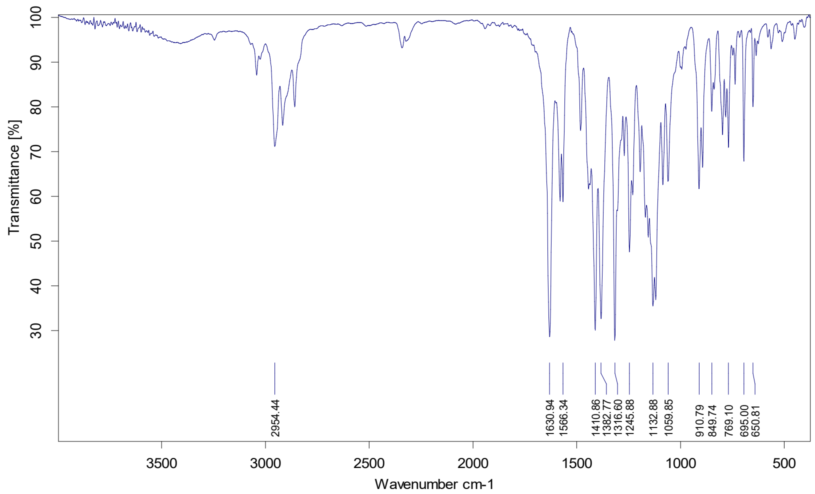


# $^1$H NMR

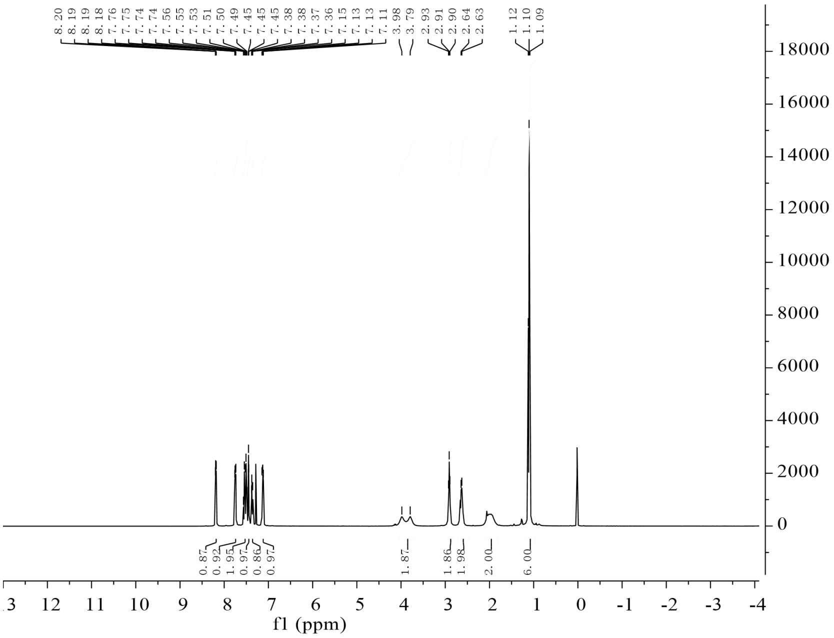

## $^{13}$C NMR

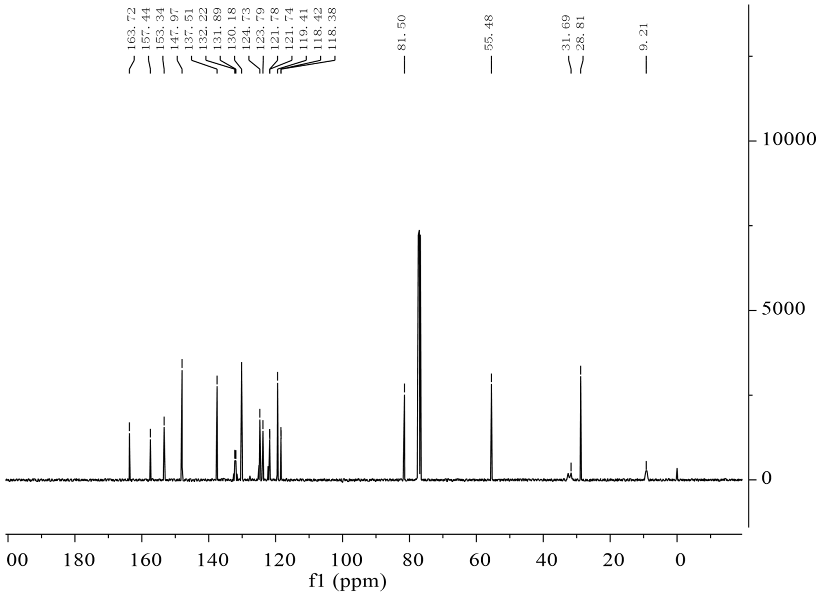


## HRMS

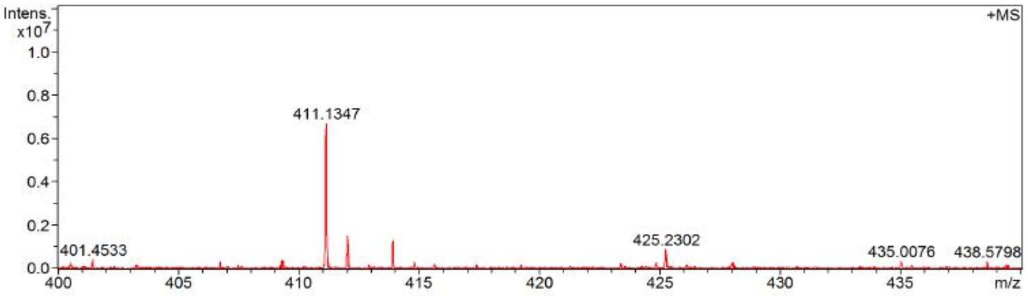


| Meas. m/z | # | Formula | m/z | err [ppm] | N-Rule | e¡¥Conf |
|---|---|---|---|---|---|---|
| 411.1347 | 1 | C 20 H 22 F 3 N 2 O 2 S | 411.1349 | 0.4 | ok | even |

White solid, m.p. 80-81 ℃; IR (KBr, cm$^{-1}$) $\nu$: 2954 (C-H), 1630 (C=O), 1566-1410 (C=C), 1245 (C-O); $^{1}$H NMR (400 MHz, $CDCl_3$) δ 8.19 (dd, $J$ = 4.9, 1.9 Hz, 1H, Py-H), 7.75 (dd, $J$ = 7.3, 2.0 Hz, 1H, Py-H), 7.59-7.47 (m, 2H, Ar-H), 7.45 (t, $J$ = 1.9 Hz, 1H, Ar-H), 7.38-7.34 (m, 1H, Ar-H), 7.13 (dd, $J$ = 7.3, 4.9 Hz, 1H, Py-H), 3.89 (d, $J$ = 75.8 Hz, 2H, N-$CH_2$), 2.91 (t, $J$ = 6.1 Hz, 2H, S-$CH_2$), 2.63-2.03 (m, 4H, -$CH_2$-), 1.10 (t, $J$ = 7.3 Hz, 6H,-C-$CH_3$); $^{13}$C NMR (101 MHz, $CDCl_3$) δ 163.72, 157.44, 153.34, 147.97, 137.51, 130.18, 124.73, 123.79, 121.78, 121.74, 119.41, 118.42, 118.38, 81.50, 55.48, 31.69, 28.81, 9.21; HRMS calcd; for [M + H$^{+}$] $C_{20}H_{22}F_3N_2O_2S$: 411.1349, found 411.1347.

# Compound 4f

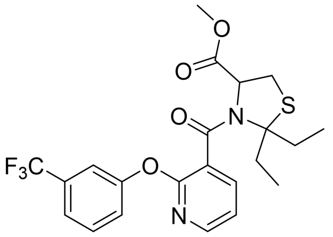


*Methyl (R)-2,2-diethyl-3-(2-(3-(trifluoromethyl)phenoxy)nicotinoyl)thiazolidine-4-carboxylate*

# IR

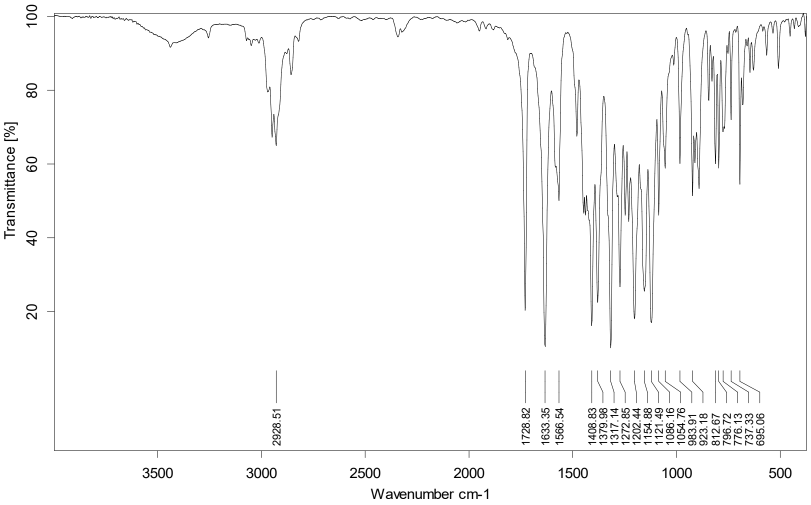


# $^1$H NMR

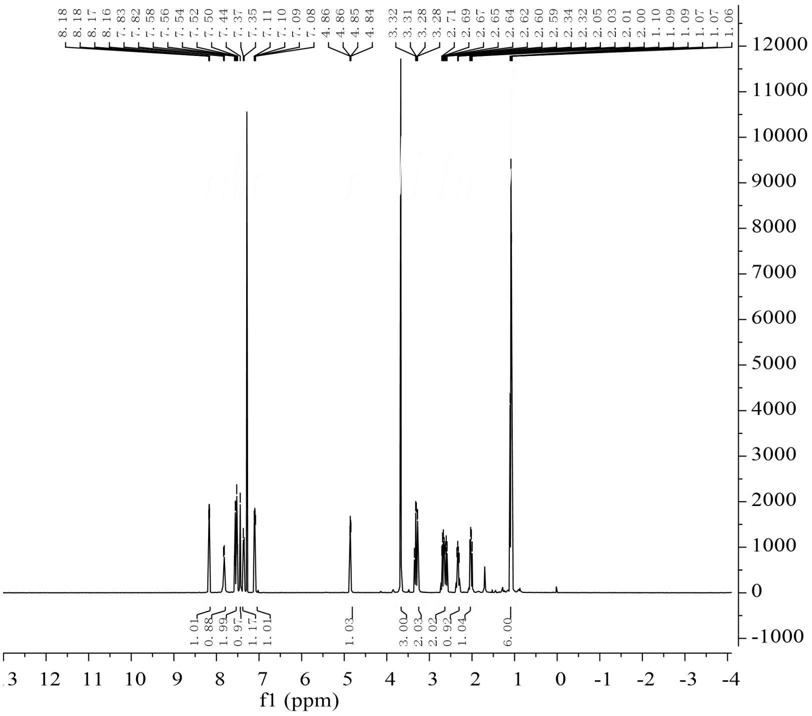

## $^{13}$C NMR

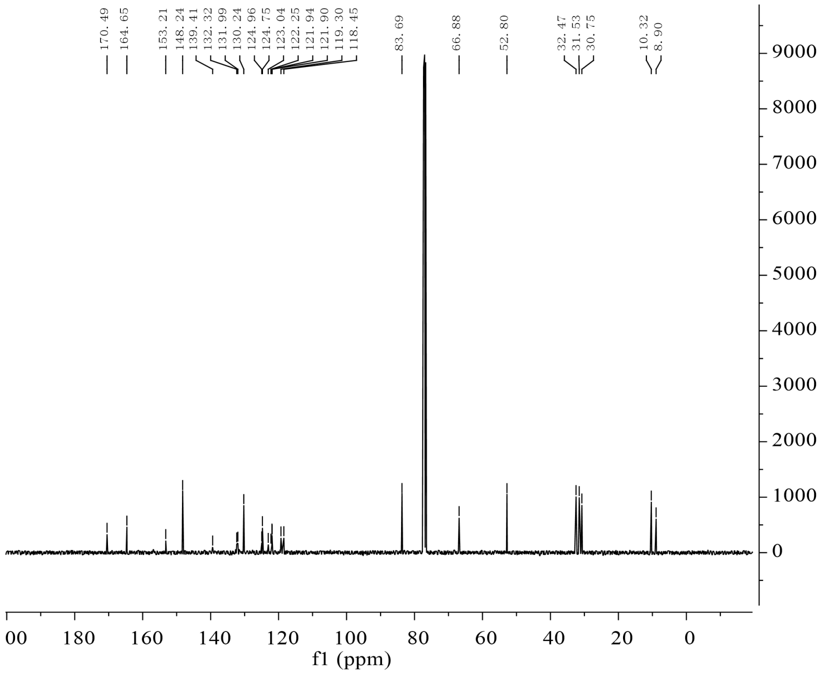


## HRMS

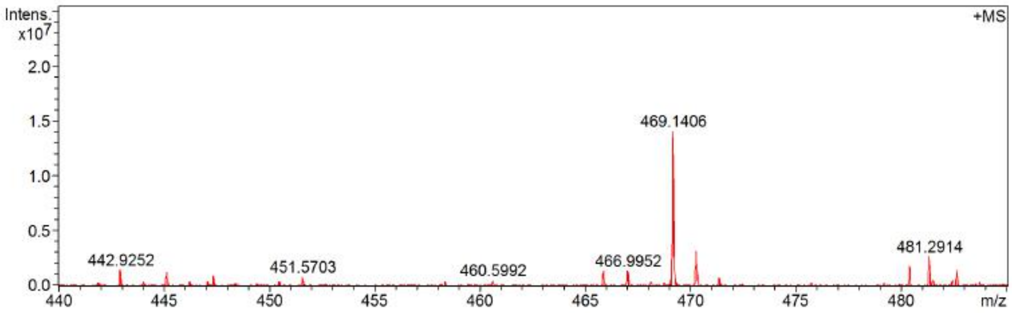


| Meas. m/z | # | Formula | m/z | err [ppm] | N-Rule | e¡¥Conf |
|---|---|---|---|---|---|---|
| 469.1406 | 1 | C 22 H 24 F 3 N 2 O 4 S | 469.1403 | -0.6 | ok | even |

White solid, m.p. 141-142 °C; IR (KBr, $cm^{-1}$) $\nu$: 2928 (C-H), 1728, 1633 (C=O), 1566-1408 (C=C), 1272 (C-O); $^1$H NMR (400 MHz, $CDCl_3$) δ 8.17 (dd, *J* = 4.9, 1.9 Hz, 1H, Py-H), 7.82 (d, *J* = 6.8 Hz, 1H, Py-H), 7.60-7.48 (m, 2H, Ar-H), 7.44 (s, 1H, Ar-H), 7.36 (d, *J* = 7.7 Hz, 1H, Ar-H), 7.10 (dd, *J* = 7.4, 4.9 Hz, 1H, Py-H), 4.85 (dd, *J* = 6.1, 2.2 Hz, 1H, N-CH), 3.67 (s, 3H, O-$CH_3$), 3.38-3.12 (m, 2H S-$CH_2$), 2.65-2.02 (m, 4H, -$CH_2$-), 1.08 (td, *J* = 7.4, 4.8 Hz, 6H, -C-$CH_3$); $^{13}$C NMR (101 MHz, $CDCl_3$) δ 170.49, 164.65, 153.21, 148.24, 130.24, 124.75, 123.04, 121.94, 121.90, 119.30, 118.45, 83.69, 66.88, 52.80, 32.47, 31.53, 30.75, 10.32, 8.90; HRMS calcd. for [M + H$^+$] $C_{22}H_{24}F_3N_2O_4S$: 469.1403, found 469.1406.

# Compound 4g

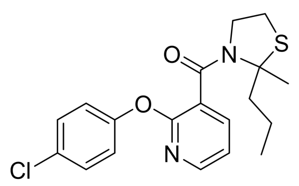

*(2-(4-chlorophenoxy)pyridin-3-yl)(2-methyl-2-propylthiazolidin-3-yl)methanone*

# IR

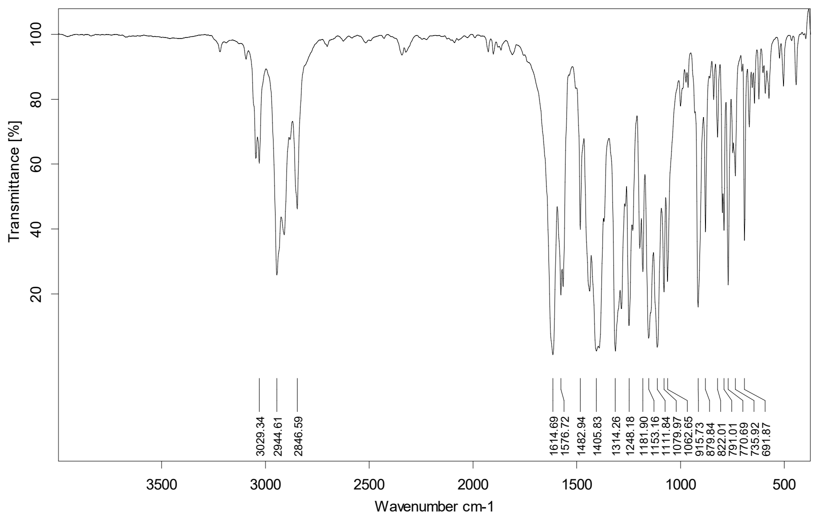


# $^{1}$H NMR

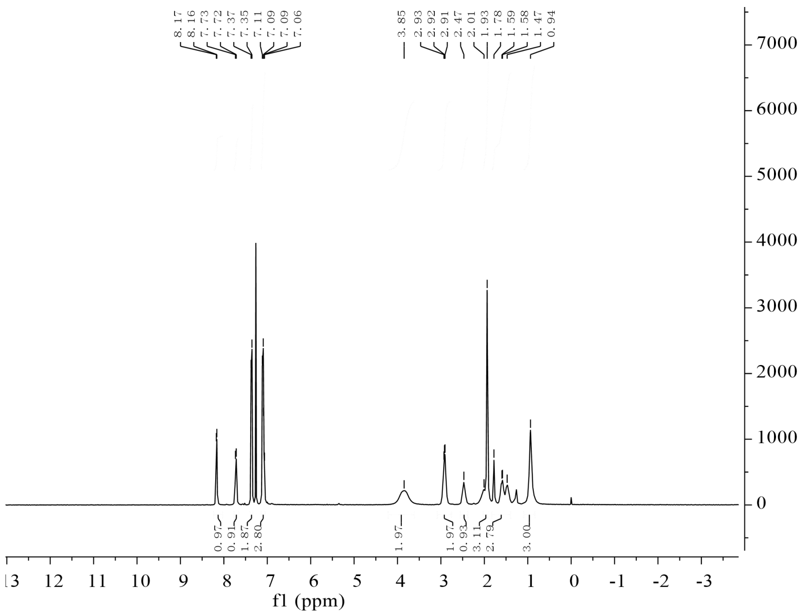

## $^{13}$C NMR

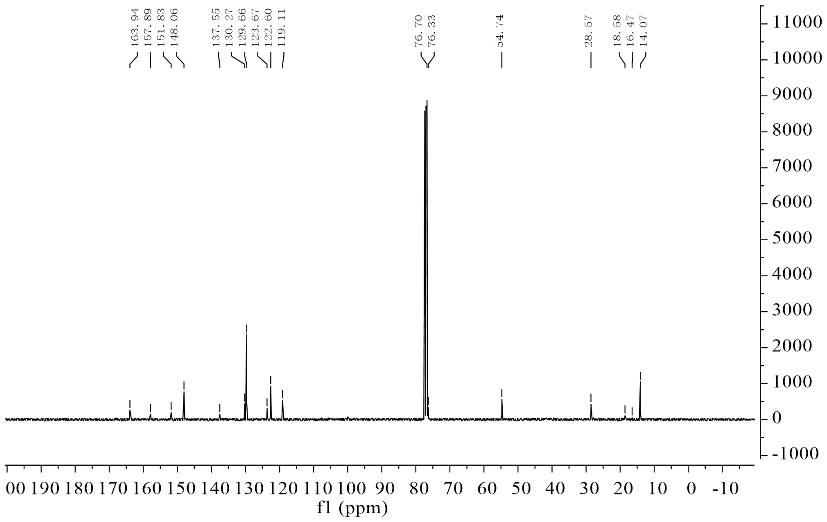

## HRMS

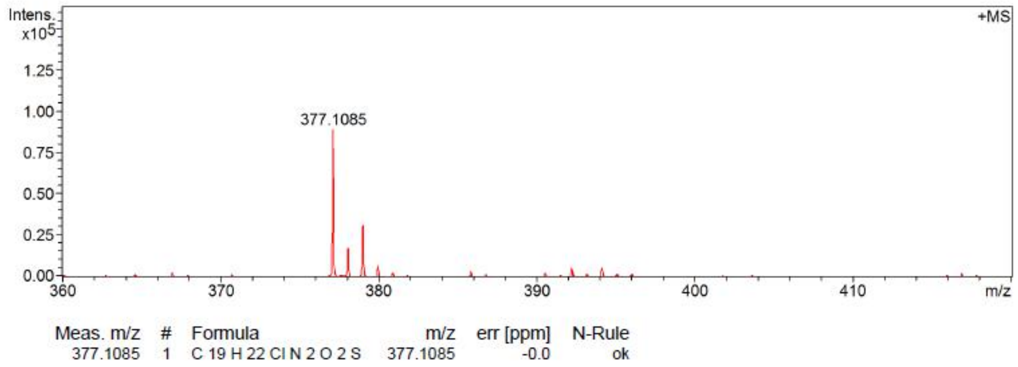

White solid, m.p. 75-76 ℃; IR (KBr, cm$^{-1}$) $\nu$: 3029-2846 (C-H), 1614 (C=O), 1576-1482 (C=C), 1248 (C-O); $^1$H NMR (400 MHz, $CDCl_3$) δ 8.17 (d, $J$ = 4.7 Hz, 1H, Py-H), 7.72 (d, $J$ = 7.0 Hz, 1H, Py-H), 7.36 (d, $J$ = 8.3 Hz, 2H, Ar-H), 7.09 (dd, $J$ = 13.4, 6.6 Hz, 3H, Ar-H, Py-H), 3.84 (s, 2H, N-$CH_2$), 3.04 -2.80 (m, 2H, S-$CH_2$), 2.47-1.93 (m, 4H, C-$CH_2$-$CH_2$-), 1.82-1.26 (t, 3H, -C-$CH_3$), 0.94 (s, 3H, -$CH_3$); $^{13}$C NMR (101 MHz, $CDCl_3$) δ 163.94, 157.89, 151.83, 148.06, 137.55, 130.27, 129.66, 123.67, 122.60, 119.11, 54.74, 28.57, 18.58, 14.07; HRMS calcd. for [M + H$^+$] $C_{19}H_{22}ClN_2O_2S$: 377.1085, found 377.1085.

# Compound 4h

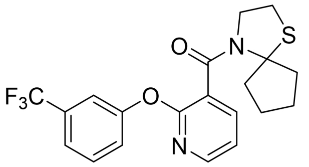


*(1-thia-4-azaspiro[4,4]nonan-4-yl)(2-(3-(trifluoromethyl)phenoxy)pyridin-3-yl)methanone*

# IR

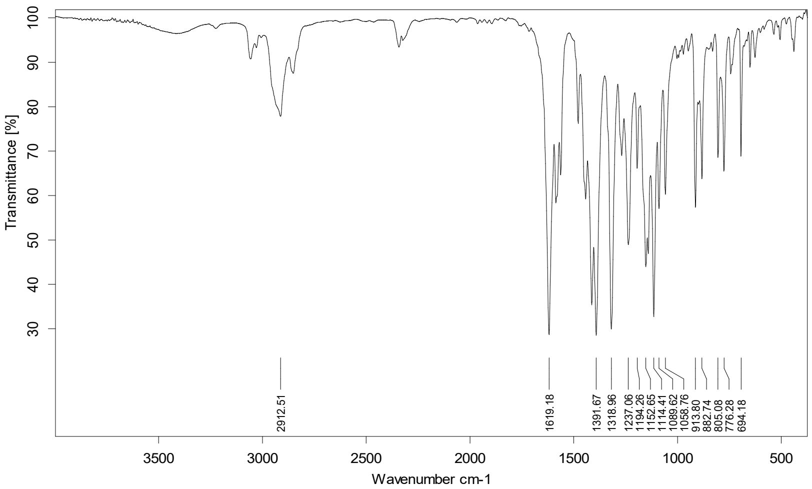


# $^{1}$H NMR

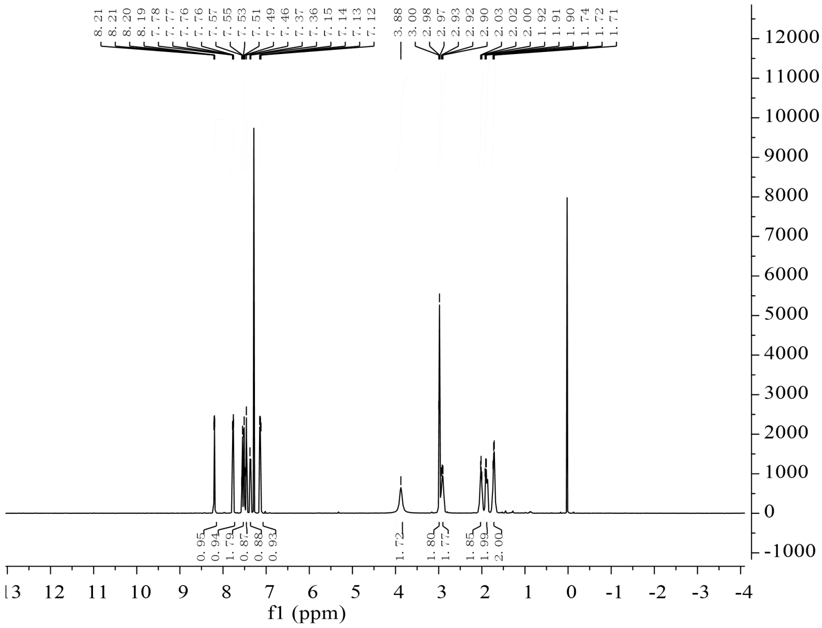

# $^{13}$C NMR

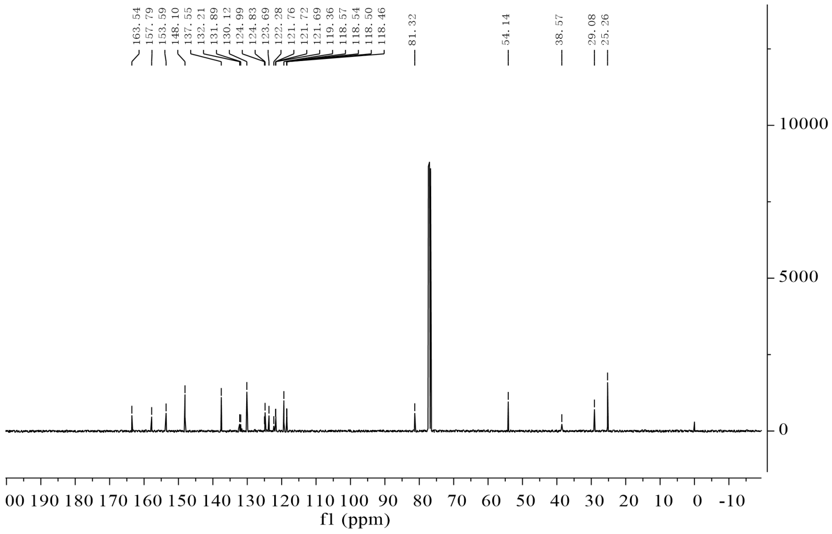


# HRMS

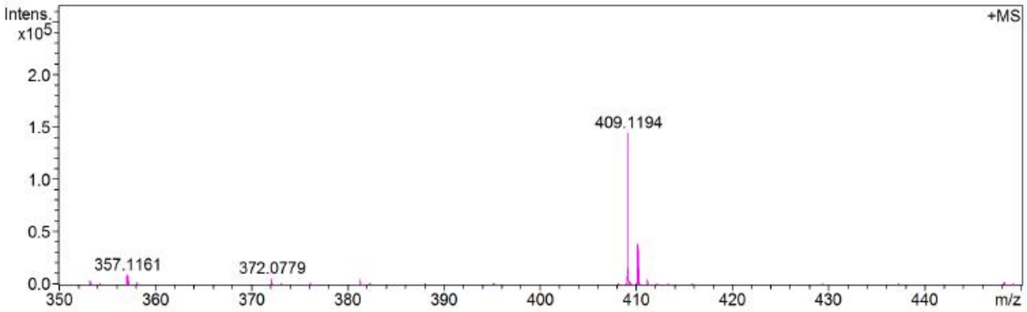


| Meas. m/z | # | Formula | m/z | err [ppm] | N-Rule | ej¥Conf |
|---|---|---|---|---|---|---|
| 409.1194 | 1 | C 20 H 20 F 3 N 2 O 2 S | 409.1192 | -0.5 | ok | even |

White solid, m.p. 102-103 °C; IR (KBr, cm$^{-1}$) $v$: 2912 (C-H), 1619 (C=O), 1571-1391 (C=C), 1237 (C-O); $^{1}$H NMR (400 MHz, $CDCl_3$) δ 8.20 (dd, $J$ = 4.9, 1.9 Hz, 1H, Py-H), 7.77 (dd, $J$ = 7.4, 1.9 Hz, 1H, Py-H), 7.53 (dt, $J$ = 14.7, 7.8 Hz, 2H, Ar-H), 7.46 (s, 1H, Ar-H), 7.37 (d, $J$ = 7.6 Hz, 1H, Ar-H), 7.13 (dd, $J$ = 7.4, 4.9 Hz, 1H, Py-H), 3.88 (s, 2H, N-$CH_2$), 2.98 (t, $J$ = 6.0 Hz, 2H, S-$CH_2$), 2.96-2.01 (m, 4H, -$CH_2$-), 1.91-1.66 (m, 4H, -$CH_2$-); $^{13}$C NMR (101 MHz, $CDCl_3$) δ 163.54, 157.79, 153.59, 148.10, 137.55, 130.12, 124.83, 123.69, 121.76, 121.72, 119.36, 118.54, 118.50, 81.32, 54.14, 38.57, 29.08, 25.26; HRMS calcd. for [M + H$^+$] $C_{20}H_{20}F_3N_2O_2S$: 409.1192, found 409.1194.

# Compound 4i

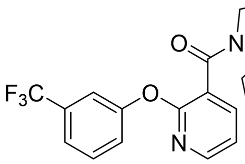


*(1-thia-4-azaspiro[4,5]decan-4-yl)(2-(3-(trifluoromethyl)phenoxy)pyridin-3-yl)methanone*

# IR

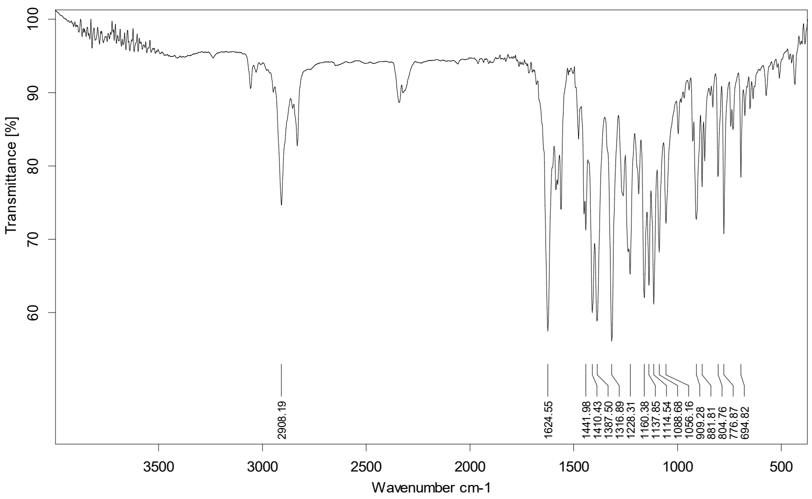


# $^1$H NMR

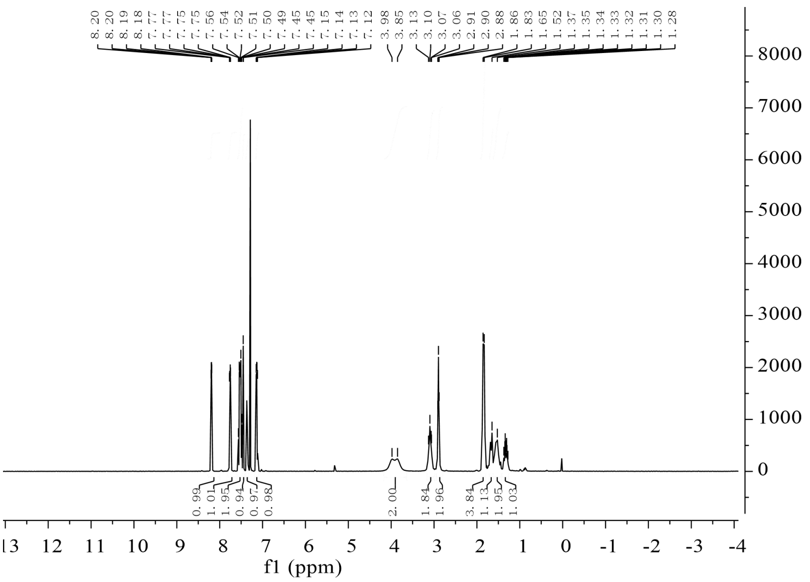

## $^{13}C$ NMR

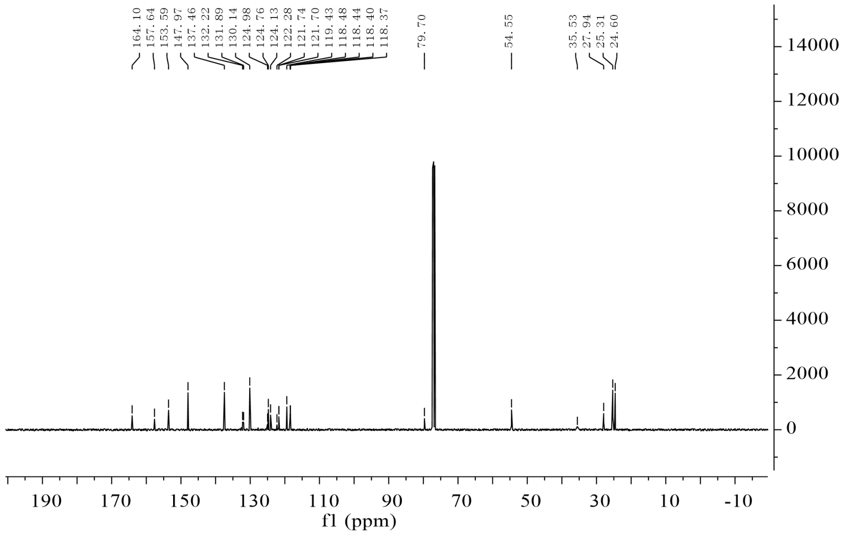


## HRMS

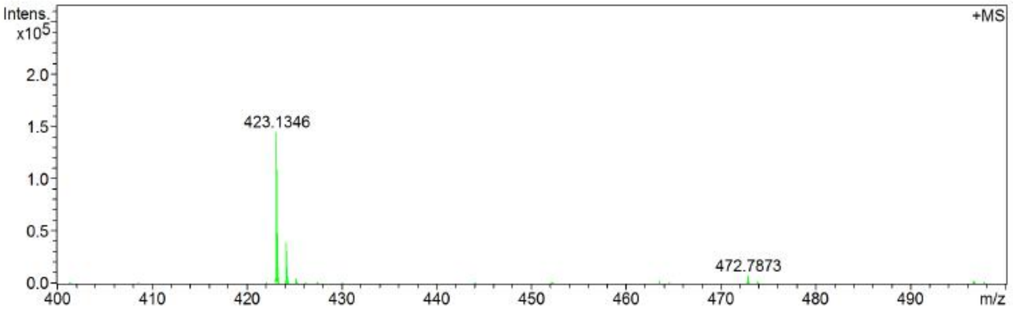


| Meas. m/z | # | Formula | m/z | err [ppm] | N-Rule | e¦¥Conf |
|---|---|---|---|---|---|---|
| 423.1346 | 1 | C 21 H 22 F 3 N 2 O 2 S | 423.1349 | 0.6 | ok | even |

White solid, m.p. 135-136 °C; IR (KBr, $cm^{-1}$) *ν*: 2908 (C-H), 1624 (C=O), 1531-1441 (C=C), 1228 (C-O); $^{1}H$ NMR (400 MHz, $CDCl_3$) δ 8.19 (dd, *J* = 4.9, 1.9 Hz, 1H, Py-H),7.76 (dd, *J* = 7.4, 1.9 Hz, 1H, Py-H), 7.52 (dt, *J* = 15.2, 7.9 Hz, 2H, Ar-H), 7.45 (d, *J* =1.8 Hz, 1H, Ar-H), 7.36 (dt, *J* = 7.9, 1.8 Hz, 1H, Ar-H), 7.13 (dd, *J* = 7.4, 5.0 Hz, 1H, Py-H), 3.92 (d, *J* = 51.7 Hz, 2H, $NCH_2$), 3.16-2.99 (m, 2H, S-$CH_2$), 2.90-1.26 (m, 10 H, -$C_5H_{10}$); $^{13}$CNMR (101 MHz, $CDCl_3$) δ 164.10 , 157.64, 153.59, 147.97, 137.46, 130.14, 124.76, 124.13, 121.74, 121.70, 119.43, 118.44, 118.40, 54.55, 35.53, 27.94, 25.31, 24.60; HRMS calcd. for [M + $H^+$] $C_{21}H_{22}F_3N_2O_2S$: 423.1349, found 423.1346.

# Compound 4j

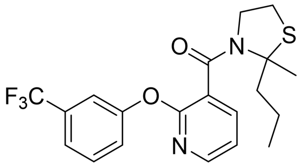


*(1-methyl-2-propylthiazolidin-3-yl)(2-(3-(trifluoromethyl)phenoxy)pyridin-3-yl)methanone*

# IR

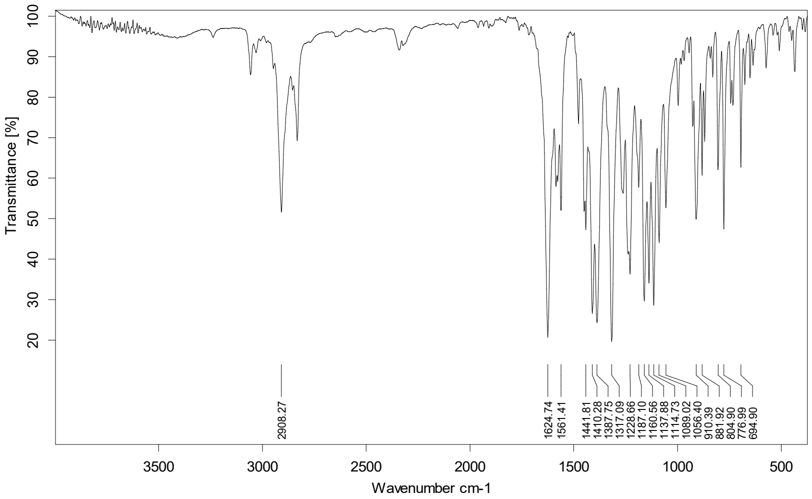


# $^{1}$H NMR

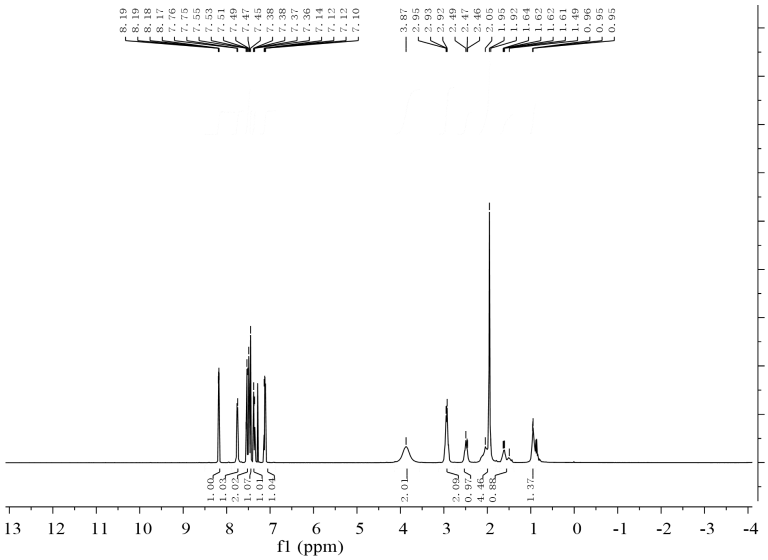

# $^{13}$C NMR

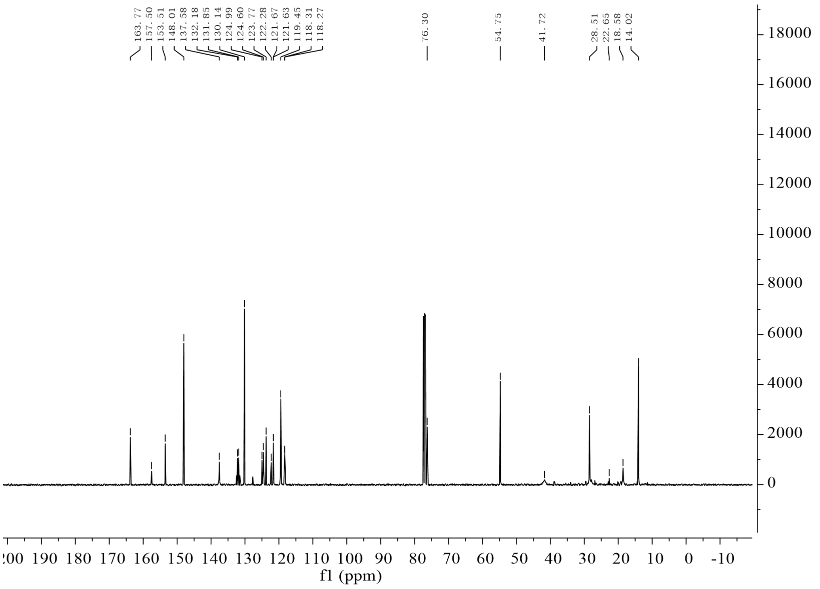


# HRMS

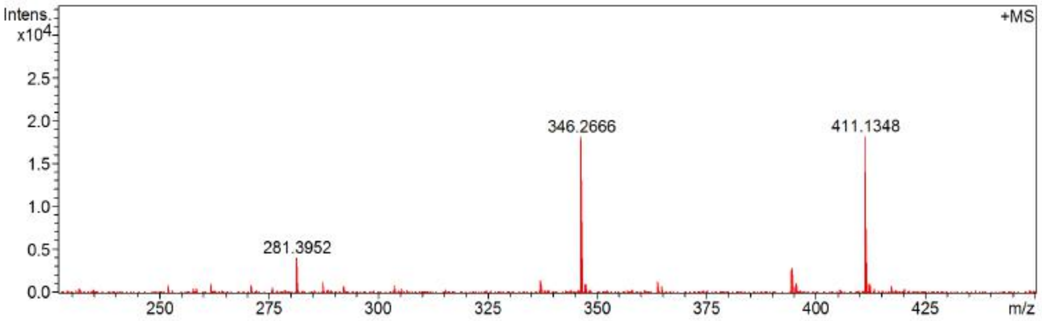


| Meas. m/z | # | Formula | m/z | err [ppm] | N-Rule |
|---|---|---|---|---|---|
| 411.1348 | 1 | C 20 H 22 F 3 N 2 O 2 S | 411.1349 | 0.1 | ok |

White solid, m.p. 89-90 °C; IR (KBr, $cm^{-1}$) $\nu$: 2908 (C-H), 1624 (C=O), 1561-1441 (C=C), 1228 (C-O); $^{1}$H NMR (400 MHz, $CDCl_3$) δ 8.18 (dd, *J* = 5.0, 1.8 Hz, 1H, Py-H), 7.75 (d, *J* = 6.3 Hz, 1H, Py-H), 7.51 (dt, *J* = 16.7, 7.9 Hz, 2H, Ar-H), 7.45 (s, 1H, Ar-H), 7.41 – 7.34 (m, 1H, Ar-H), 7.12 (dd, *J* = 7.3, 4.9 Hz, 1H, Py-H), 3.87 (s, 2H, N-$CH_2$), 3.12 – 2.74 (m, 2H, S-$CH_2$), 2.48-1.62 (m, 7H, -$CH_2$-$CH_2$-$CH_3$), 0.95 (s, *J* = 2.4 Hz, 3H, -$CH_3$); $^{13}$C NMR (101 MHz, $CDCl_3$) δ 163.77, 157.50, 153.51, 148.01, 137.58, 130.14, 124.60, 123.77, 121.67, 121.63, 119.45, 118.31, 76.30, 54.75, 41.72, 28.51, 18.58, 14.02; HRMS calcd. for [M + $H^+$] $C_{20}H_{22}F_3N_2O_2S$: 411.1349, found 411.1348.

# Compound 4k

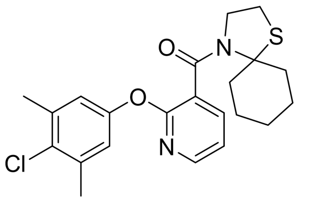

*(2-(4-chloro-3,5-dimethylphenoxy)pyridin-3-yl)(1-thia-4-azaspiro[4.5]decan-4-yl)methanone*

# IR

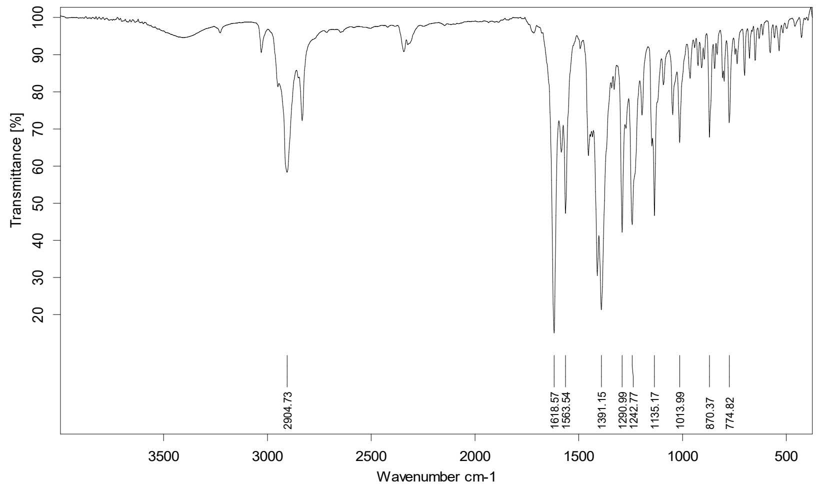


# $^{1}$H NMR

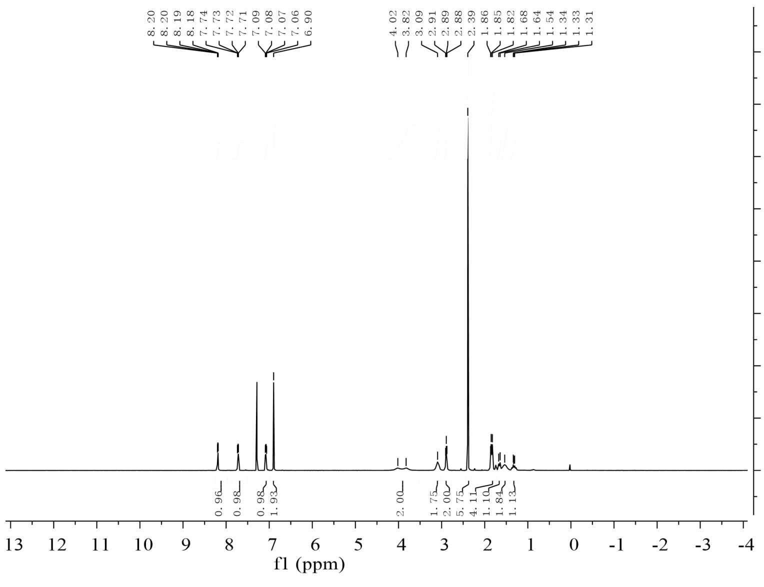

# $^{13}$C NMR

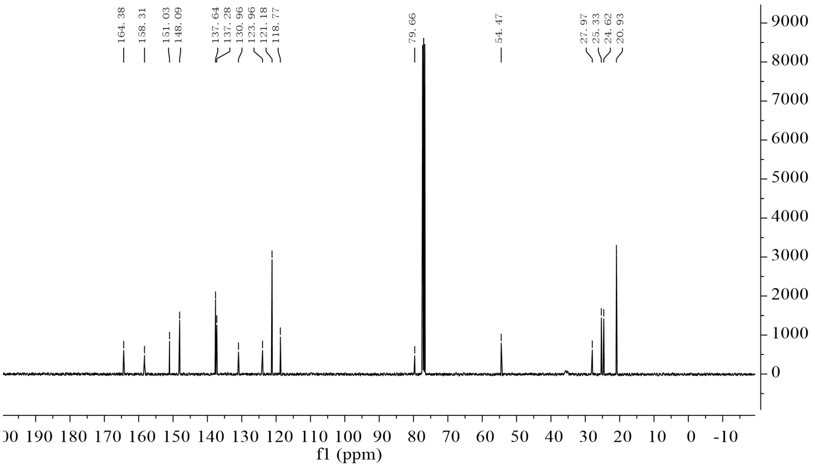


# HRMS

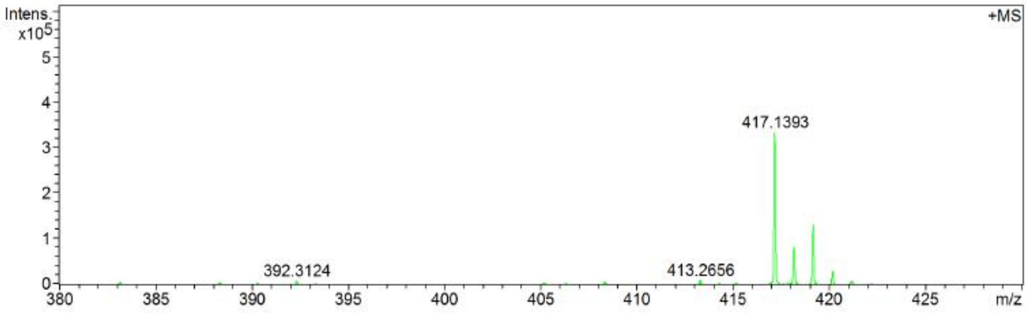


| Meas. m/z | # | Formula | m/z | err [ppm] | N-Rule |
|---|---|---|---|---|---|
| 417.1393 | 1 | C 22 H 26 Cl N 2 O 2 S | 417.1398 | 1.1 | ok |

White solid, m.p. 149-150 ℃; IR (KBr, $cm^{-1}$) *ν*: 2904 (C-H), 1618 (C=O), 1563-1391 (C=C), 1242 (C-O); $^1$H NMR (400 MHz, $CDCl_3$) δ 8.19 (dd, *J* = 5.0, 1.9 Hz, 1H, Py-H), 7.73 (dd, *J* = 7.3, 1.9 Hz, 1H, Py-H), 7.07 (dd, *J* = 7.3, 4.9 Hz, 1H, Ar-H), 6.90 (s, 2H, Ar-H, Py-H), 3.92 (d, *J* = 77.0 Hz, 2H, N-$CH_2$), 3.09 (s, 2H, S-$CH_2$), 2.89 (t, *J* = 5.9 Hz, 2H, -$CH_2$), 2.39 (s, 6H, Ar-$CH_3$), 1.90-1.32 (m, 8H, -$C_4H_8$); $^{13}$C NMR (101 MHz, $CDCl_3$) δ 164.38, 158.31, 151.03, 148.09, 137.64, 137.28, 123.96, 121.18, 118.77, 79.66, 54.47, 27.97, 25.33, 24.62, 20.93; HRMS calcd. for [M + $H^+$] $C_{22}H_{26}ClN_2O_2S$: 417.1398, found 417.1393.

# Compound 4l

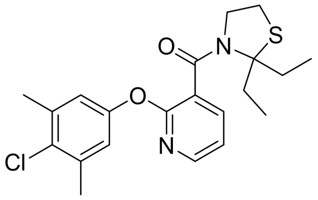

*(2-(4-chloro-3,5-dimethylphenoxy)pyridin-3-yl)(2,2-diethylthiazolidin-3-yl)methanone*

# IR

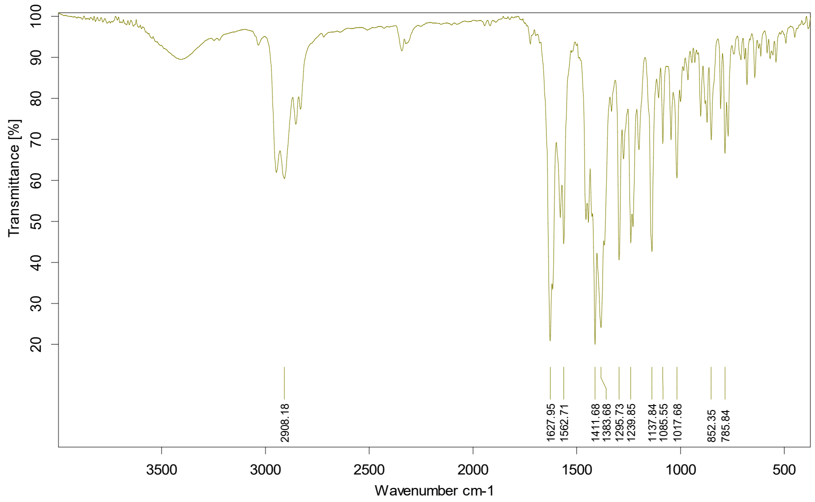


# $^{1}$H NMR

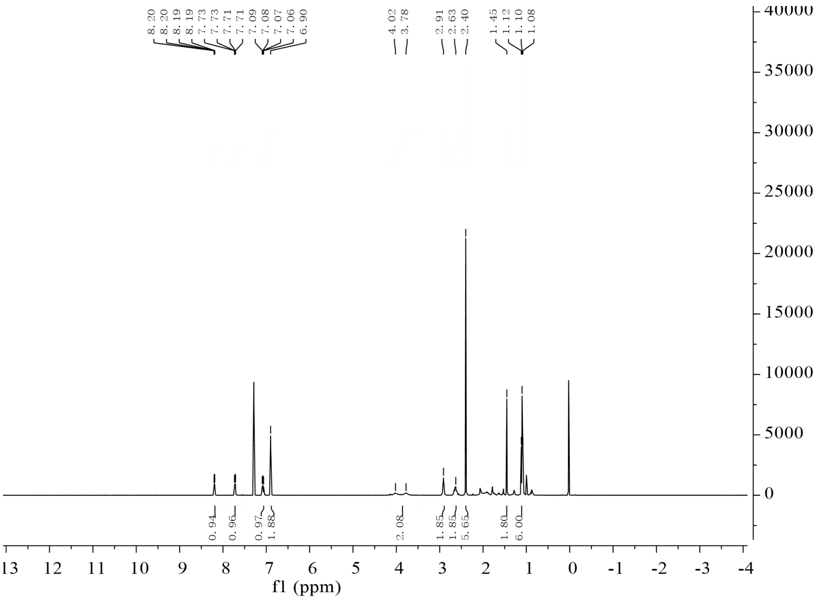

## $^{13}$C NMR

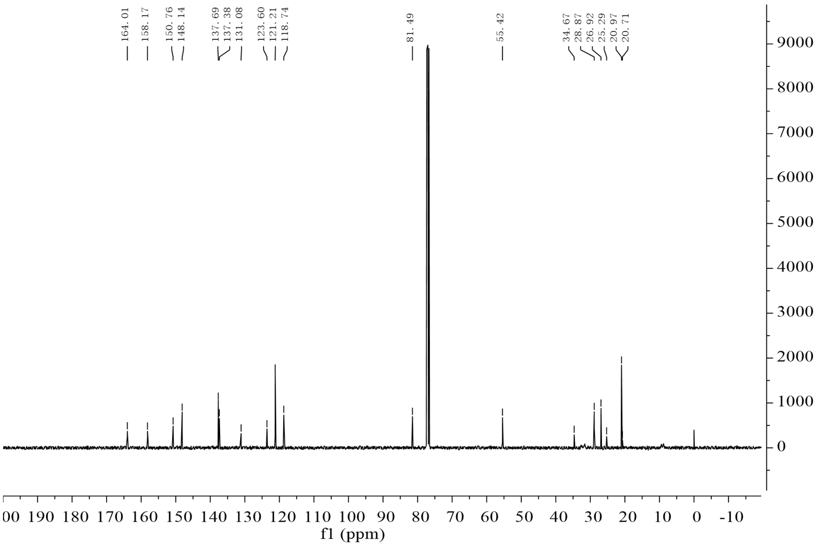


## HRMS

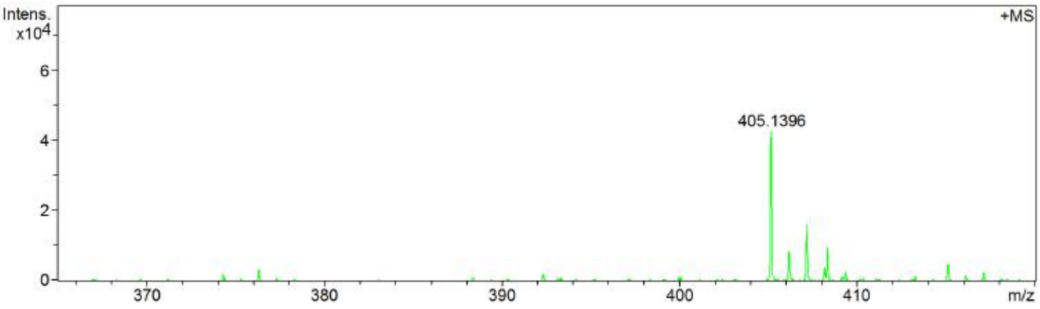


| Meas. m/z | # | Formula | m/z | err [ppm] | N-Rule |
|---|---|---|---|---|---|
| 405.1396 | 1 | C 21 H 26 Cl N 2 O 2 S | 405.1398 | 0.5 | ok |

White solid, m.p. 129-130 ℃; IR (KBr, cm$^{-1}$) *ν*: 2908 (C-H), 1627 (C=O), 1562-1411 (C=C), 1239 (C-O); $^1$H NMR (400 MHz, $CDCl_3$) δ 8.20 (dd, *J* = 5.0, 1.8 Hz, 1H, Py-H), 7.72 (dd, *J* = 7.4, 1.8 Hz, 1H, Py-H), 7.08 (dd, *J* = 7.3, 4.9 Hz, 1H, Ar-H), 6.90 (s, 2H, Ar-H, Py-H), 3.90 (d, *J* = 97.4 Hz, 2H, N-$CH_2$), 2.91 (s, 2H, S-$CH_2$), 2.63 (s, 2H, -$CH_2$-), 2.40 (s, 6H, Ar-$CH_3$), 1.45 (s, 2H, -$CH_2$-), 1.10 (t, *J* = 7.3 Hz, 6H, -C-$CH_3$); $^{13}$C NMR (101 MHz, $CDCl_3$) δ 164.01, 158.17, 150.76, 148.14, 137.69, 137.38, 131.08, 123.60, 121.21, 118.74, 81.49, 55.42, 28.87, 26.92, 20.97; HRMS calcd. for [M + H$^+$] $C_{21}H_{26}ClN_2O_2S$: 405.1398, found 405.1396.

# Compound 4m

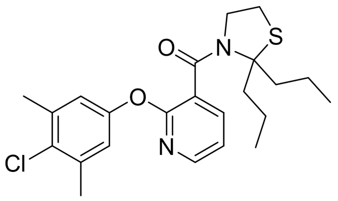


*(2-(4-chloro-3,5-dimethylphenoxy)pyridin-3-yl)(2,2-dipropylthiazolidin-3-yl)methanone*

# IR

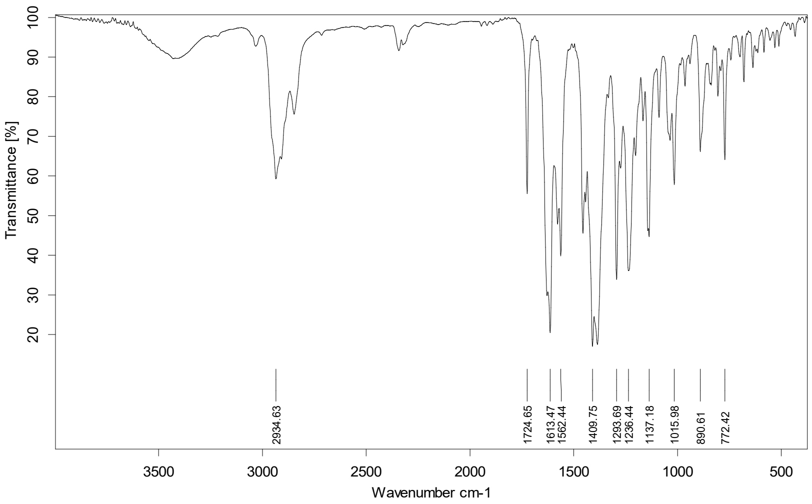


# $^1$H NMR

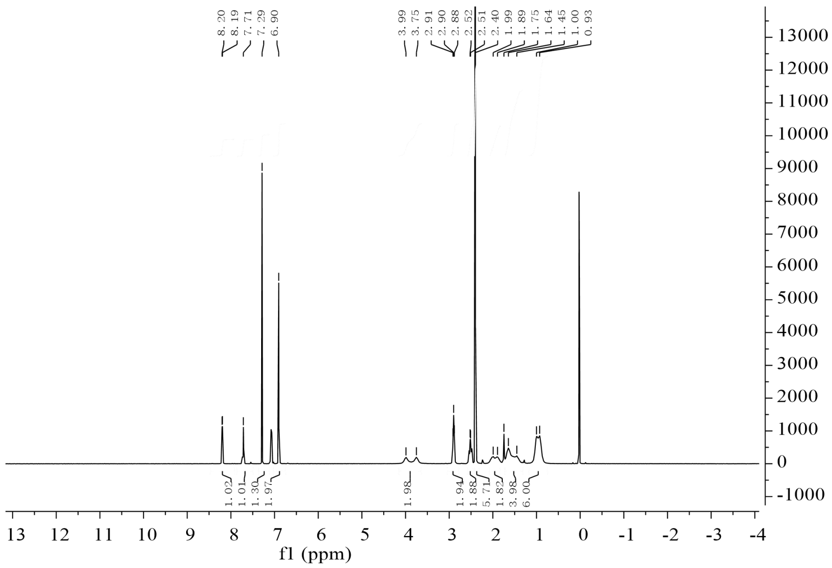

## $^{13}$C NMR

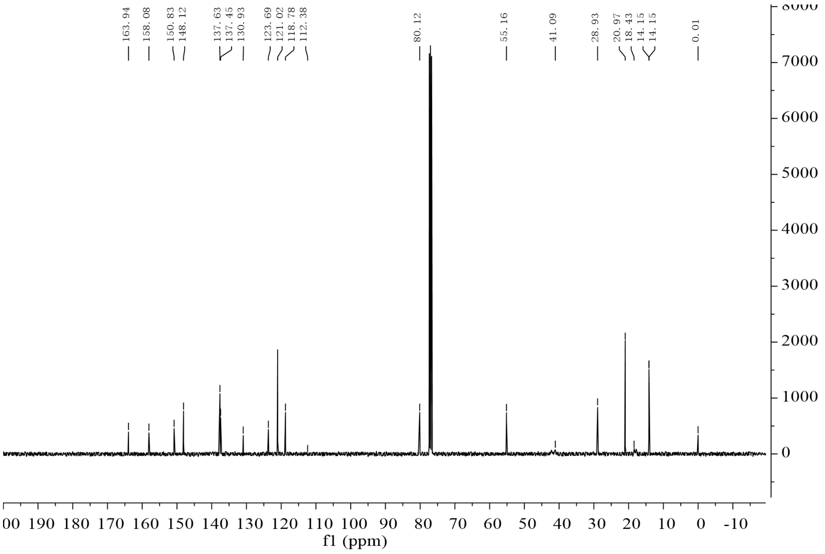


## HRMS

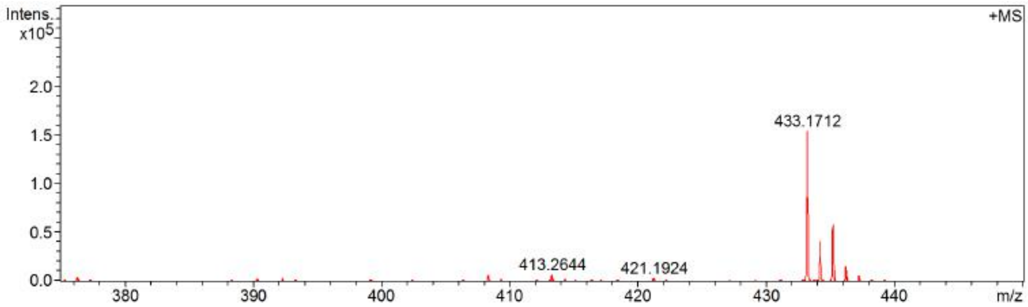


| Meas. m/z | # | Formula | m/z | err [ppm] | N-Rule |
|---|---|---|---|---|---|
| 433.1712 | 1 | C 23 H 30 Cl N 2 O 2 S | 433.1711 | -0.2 | ok |

White solid, m.p. 135-136 ℃; IR (KBr, cm$^{-1}$) $\nu$: 2934 (C-H), 1613 (C=O), 1562-1409 (C=C), 1236 (C-O); $^{1}$H NMR (400 MHz, $CDCl_3$) δ 8.20 (d, $J$ = 3.1 Hz, 1H, Py-H), 7.71 (s, 1H, Py-H), 7.29 (s, 1H, Ar-H), 6.90 (s, 2H, Ar-H, Py-H), 3.87 (d, $J$ = 95.3 Hz, 2H, N-$CH_2$), 2.90 (t, $J$ = 6.0 Hz, 2H, S-$CH_2$), 2.52 (t, $J$ = 3.9 Hz, 2H, -$CH_2$-), 2.40 (s, 6H, Ar-$CH_3$), 1.94 (t, $J$ = 39.7 Hz, 2H, -$CH_2$-), 1.64 (s, 4H, -C-$CH_2$-), 0.96 (d, $J$ = 28.9 Hz, 6H,-C-$CH_3$); $^{13}$C NMR (101 MHz, $CDCl_3$) δ 163.94, 158.08, 148.12, 137.63, 137.45, 123.69, 121.02, 118.78, 80.12, 55.16, 41.09, 28.93, 20.97, 14.15, 14.15; HRMS calcd. for [M + H$^+$] $C_{23}H_{30}ClN_2O_2S$: 433.1711, found 433.1712.

# Compound 4n

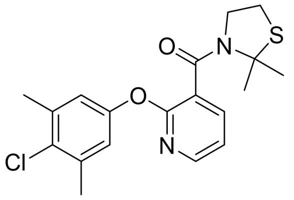

*(2-(4-chloro-3,5-dimethylphenoxy)pyridin-3-yl)(2,2-dimethylthiazolidin-3-yl)methanone*

# IR

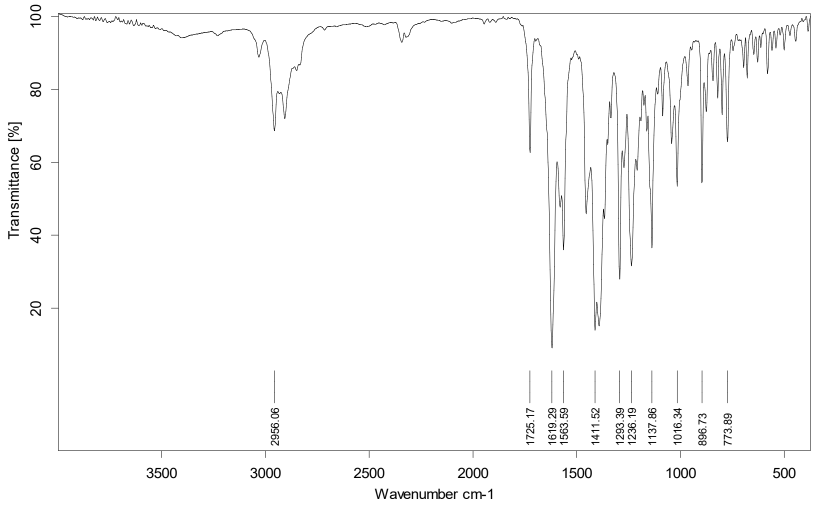

# $^{1}$H NMR

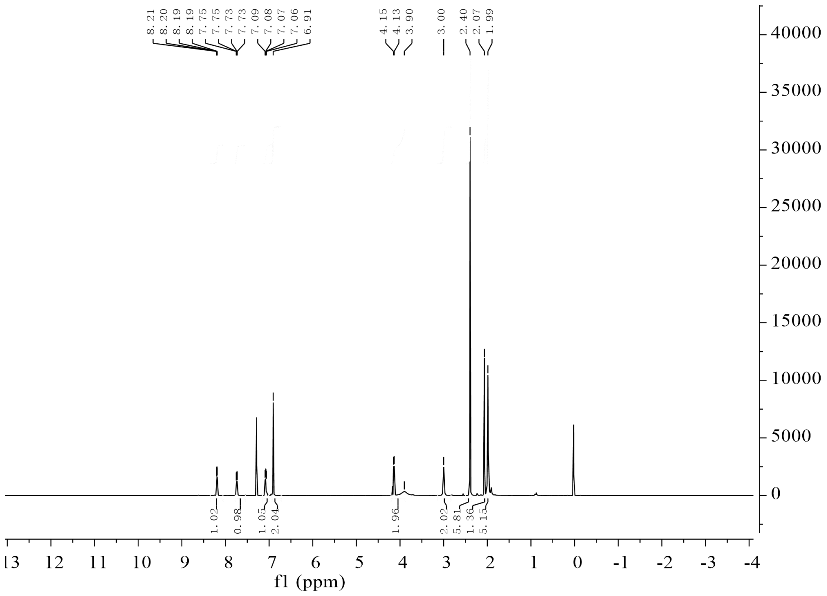

## $^{13}$C NMR

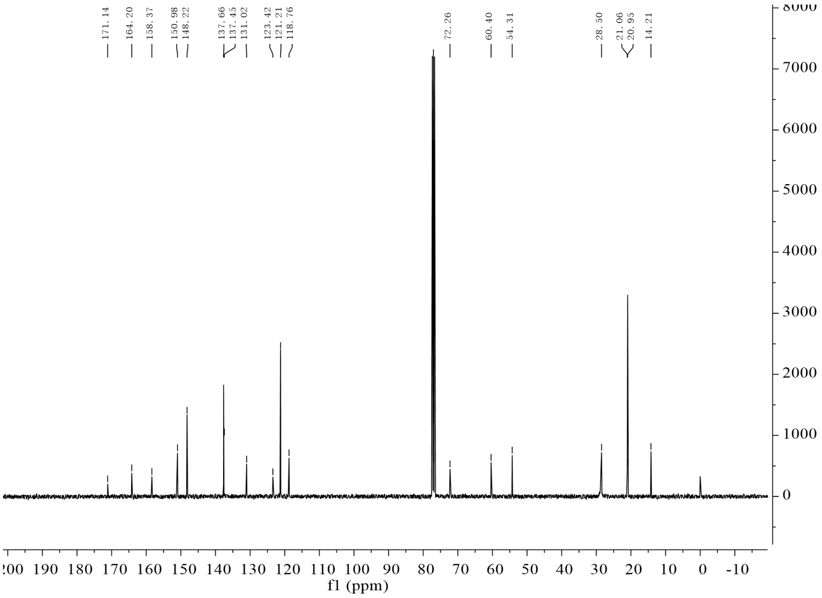


## HRMS

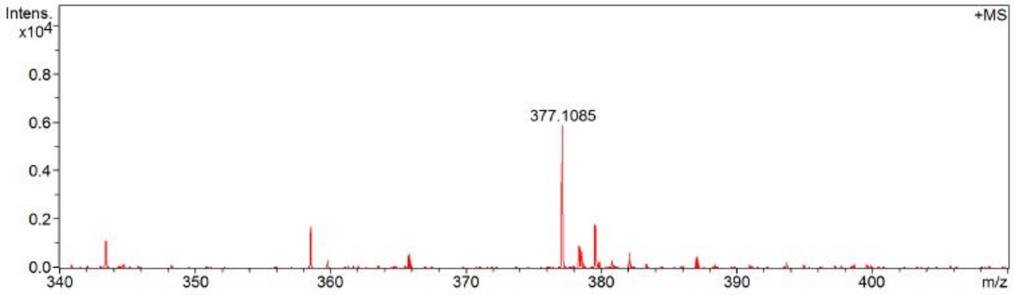


| Meas. m/z | # | Formula | m/z | err [ppm] | N-Rule |
|---|---|---|---|---|---|
| 377.1085 | 1 | C 19 H 22 Cl N 2 O 2 S | 377.1085 | 0.0 | ok |

White solid, m.p. 124-126 °C; IR (KBr, cm$^{-1}$) $\nu$: 2956 (C-H), 1619 (C=O), 1563-1441 (C=C), 1236 (C-O); $^{1}$H NMR (400 MHz, $CDCl_3$) δ 8.20 (dd, *J* = 5.0, 1.9 Hz, 1H, Py-H), 7.74 (dd, *J* = 7.4, 1.8 Hz, 1H, Py-H), 7.08 (dd, *J* = 7.3, 4.9 Hz, 1H, Ar-H), 6.91 (s, 2H, Ar-H, Py-H), 4.20 – 3.89 (m, 2H, N-$CH_2$), 3.00 (s, 2H, S-$CH_2$), 2.40-1.99 ( m, 6H, -$(CH_3)_2$); $^{13}$C NMR (101 MHz, $CDCl_3$) δ 164.20, 158.37, 150.98, 148.22, 137.66, 137.45, 131.02, 123.42, 121.21, 118.76, 72.26, 60.40, 54.31, 28.50, 20.95, 14.21; HRMS calcd. for [M + H$^{+}$] $C_{19}H_{22}Cl\ N_2O_2S$: 377.1085, found 377.1085.

# Compound 4o

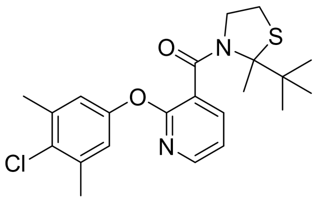

*(2-(tert-butyl)-2-methylthiazolidin-3-yl)(2-(4-chloro-3,5-dimethylphenoxy)pyridin-3-yl)methanon e*

# IR

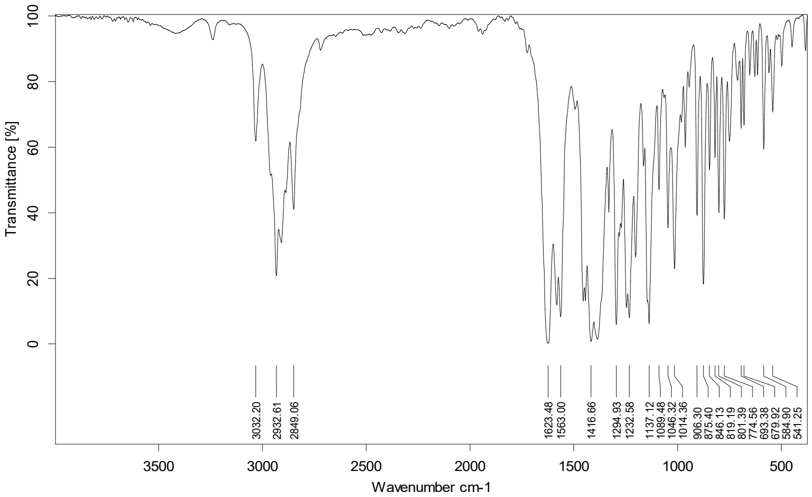

# $^1$H NMR

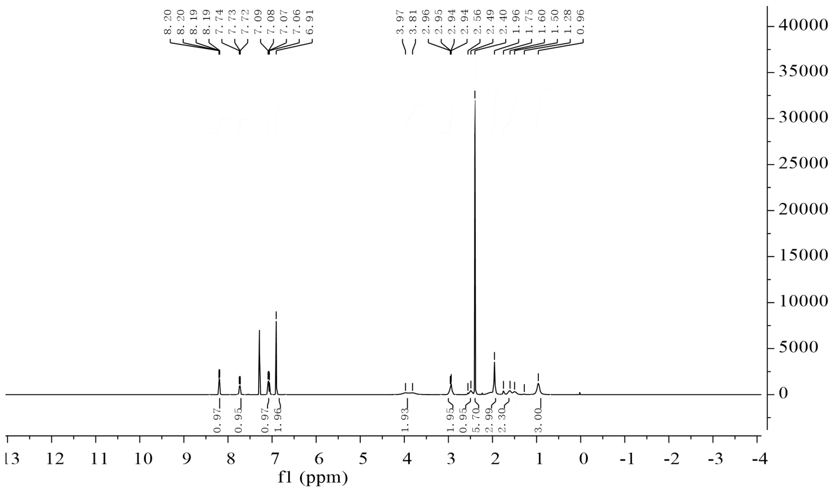

## $^{13}C$ NMR

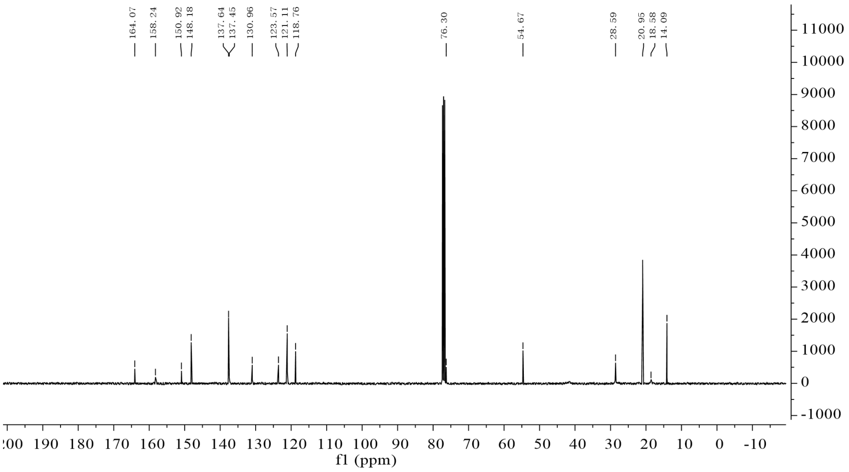

## HRMS

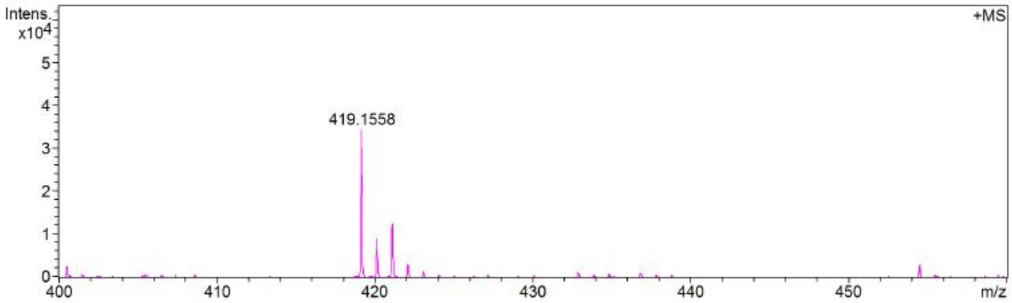


| Meas. m/z | # | Formula | m/z | err [ppm] | N-Rule |
|---|---|---|---|---|---|
| 419.1558 | 1 | C 22 H 28 Cl N 2 O 2 S | 419.1555 | -0.8 | ok |

White solid, m.p. 112-114 ℃; IR (KBr, cm$^{-1}$) $\nu$: 3032-2849 (C-H), 1623 (C=O), 1563-1416 (C=C), 1232 (C-O); $^{1}H$ NMR (400 MHz, $CDCl_3$) δ 8.20 (dd, $J$ = 4.9, 1.9 Hz, 1H, Py-H), 7.79-7.62 (m, 1H, Py-H), 7.08 (dd, $J$ = 7.3, 4.9 Hz, 1H, Ar-H), 6.91 (s, 2H, Ar-H, Py-H), 3.89 (d, $J$ = 64.2 Hz, 2H, N-$CH_2$), 3.17-2.83 (m, 2H, S-$CH_2$), 2.52-0.96 (m, 12H, -$CH_3$); $^{13}C$ NMR (101 MHz, $CDCl_3$) δ 164.07, 158.24, 150.92, 148.18, 137.64, 137.45, 123.57, 121.11, 118.76, 54.67, 28.59, 20.95, 18.58, 14.09; HRMS calcd. for [M + H$^+$] $C_{22}H_{28}ClN_2O_2S$: 419.1555, found 419.1558.

# Compound 4p

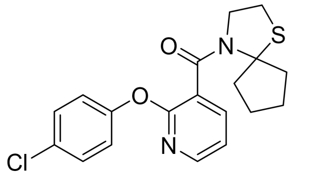


*(2-(4-chlorophenoxy)pyridin-3-yl)(1-thia-4-azaspiro[4.4]nonan-4-yl)methanone*

# IR

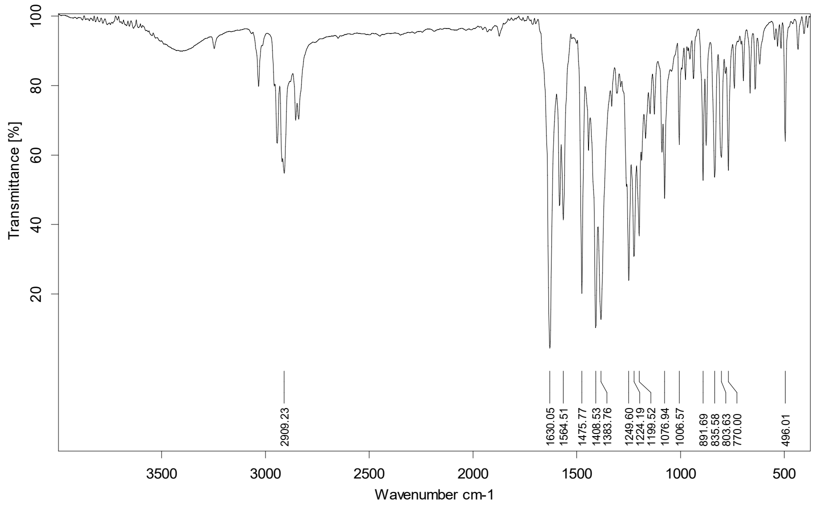


# $^1$H NMR

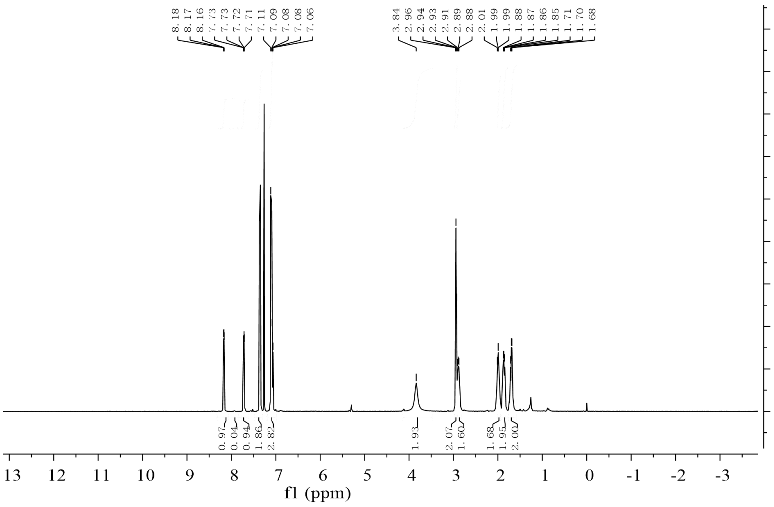

# $^{13}$C NMR

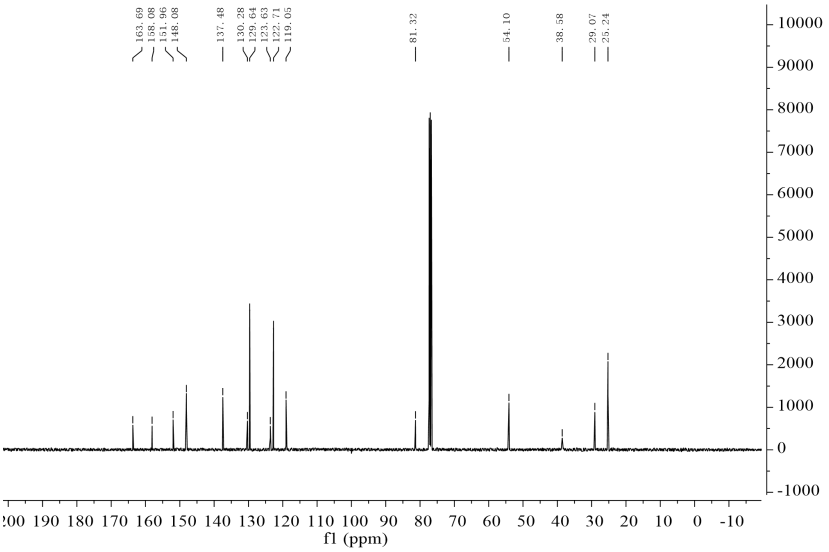


# HRMS

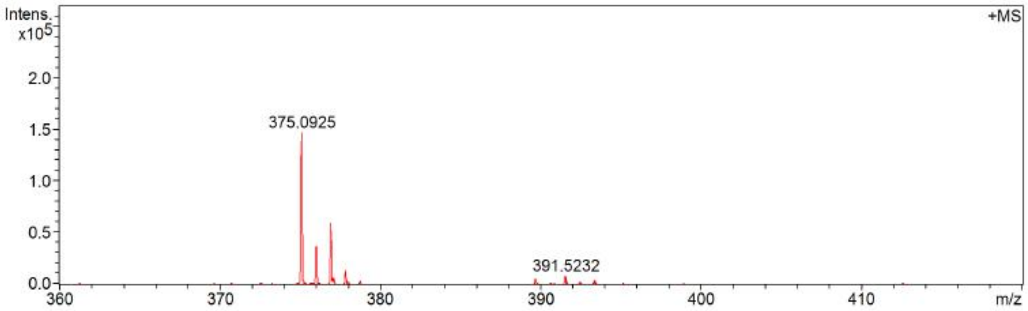


| Meas. m/z | # | Formula | m/z | err [ppm] | N-Rule |
|---|---|---|---|---|---|
| 375.0925 | 1 | C 19 H 20 Cl N 2 O 2 S | 375.0929 | 0.9 | ok |

White solid, m.p. 127-129 ℃; IR (KBr, cm$^{-1}$) $\nu$: 2909 (C-H), 1630 (C=O), 1564-1475 (C=C), 1249 (C-O); $^1$H NMR (400 MHz, $CDCl_3$) δ 8.32 – 7.92 (m, 1H, Py-H), 7.93 (d, *J* = 7.9 Hz, 1H, Py-H), 7.72 (dd, *J* = 7.4, 1.9 Hz, 1H, Ar-H), 7.47 – 7.30 (m, 2H, Ar-H), 7.19-6.99 (m, 3H, Ar-H, Py-H), 3.84 (s, 2H, N-$CH_2$), 2.94 (t, *J* = 6.0 Hz, 2H, S-$CH_2$), 2.92-1.58 (m, 8H, -$C_4H_8$); $^{13}$C NMR (101 MHz, $CDCl_3$) δ 163.69, 151.96, 148.08, 137.48, 129.64, 123.63, 122.71, 119.05, 81.32, 54.10, 38.58, 29.07, 25.24; HRMS calcd. for [M + $H^+$] $C_{19}H_{20}ClN_2O_2S$: 375.0929, found 375.0925.

# Compound 4q

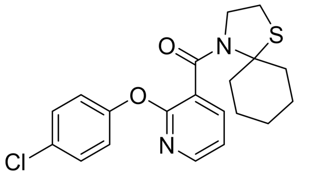


*(2-(4-chlorophenoxy)pyridin-3-yl)(1-thia-4-azaspiro[4.5]decan-4-yl)methanone*

# IR

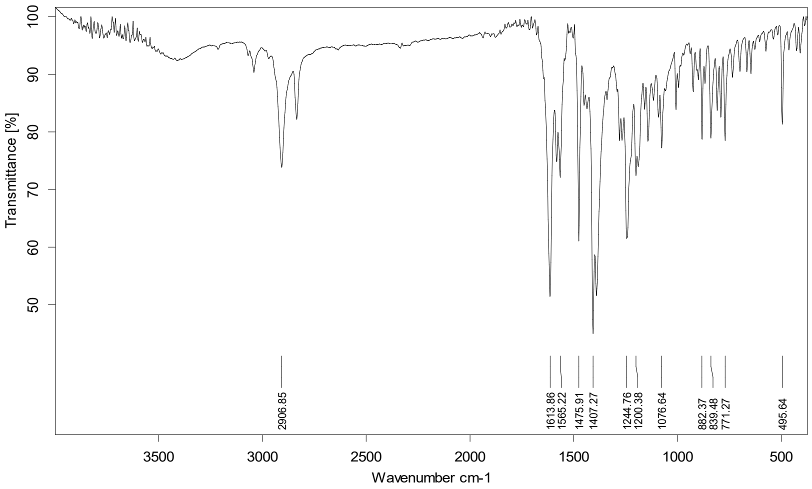


# $^{1}$H NMR

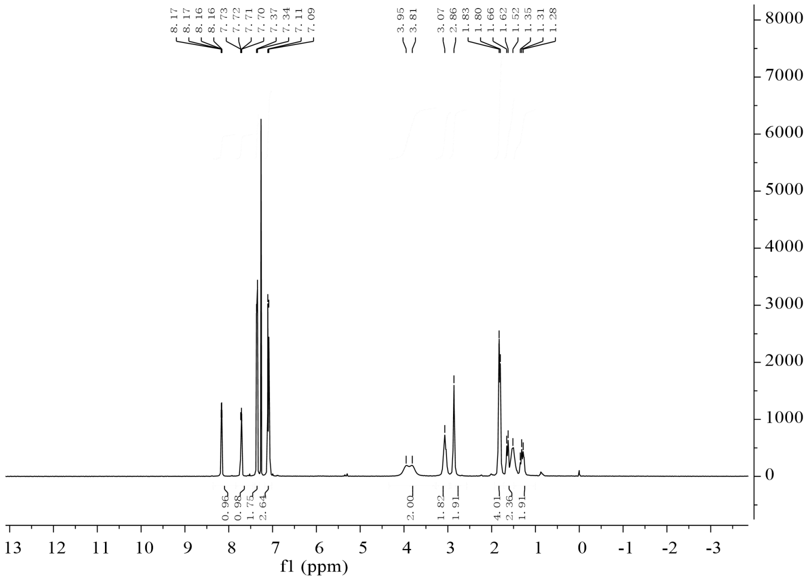

# $^{13}$C NMR

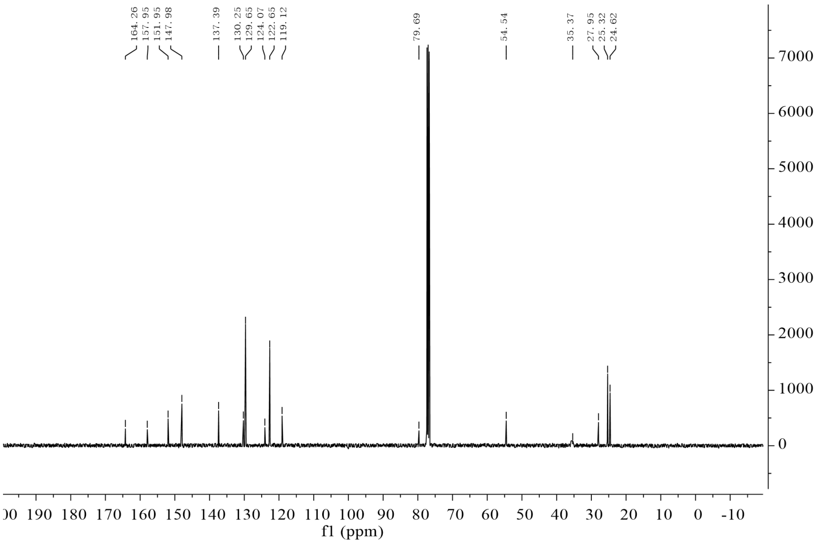


# HRMS

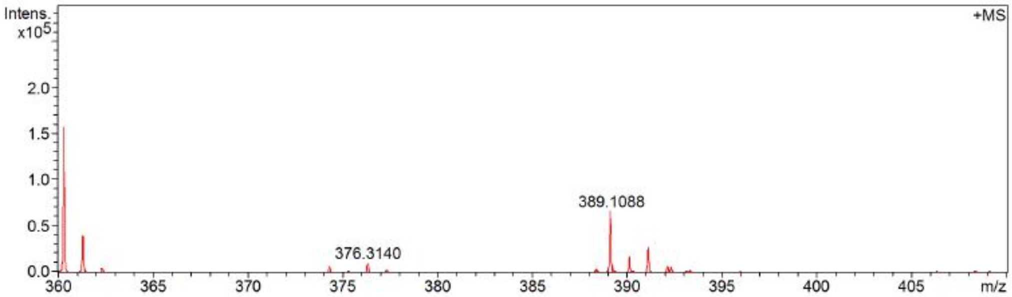


| Meas. m/z | # | Formula | m/z | err [ppm] | N-Rule |
|---|---|---|---|---|---|
| 389.1088 | 1 | C 20 H 22 Cl N 2 O 2 S | 389.1085 | -0.7 | ok |

White solid, m.p. 135-136 ℃; IR (KBr, cm$^{-1}$) *ν*: 2906 (C-H), 1613 (C=O), 1565-1475 (C=C), 1244 (C-O); $^{1}$H NMR (400 MHz, $CDCl_3$) δ 8.16 (dd, *J* = 5.0, 1.7 Hz, 1H, Py-H), 7.72 (dd, *J* = 7.3, 1.8 Hz, 1H, Py-H), 7.36 (d, *J* = 8.6 Hz, 2H, Ar-H), 7.10 (d, *J* = 8.8 Hz, 3H, Ar-H, Py-H), 3.88 (d, *J* = 56.0 Hz, 2H, N-$CH_2$), 3.07 (s, 2H, S-$CH_2$), 2.86-1.00 (m, 10H, -$C_5H_{10}$); $^{13}$C NMR (101 MHz, $CDCl_3$) δ 157.95, 151.95, 147.98, 137.39, 130.25, 129.65, 124.07, 122.65, 119.12, 54.54, 27.95, 25.32, 24.62; HRMS calcd. for [M + H$^{+}$] $C_{20}H_{22}ClN_2O_2S$: 389.1085, found 389.1088.

# Compound 4r

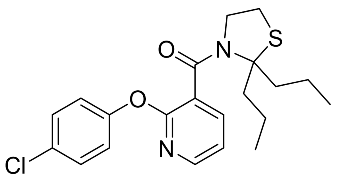

*(2-(4-chlorophenoxy)pyridin-3-yl)(2,2-dipropylthiazolidin-3-yl)methanone*

# IR

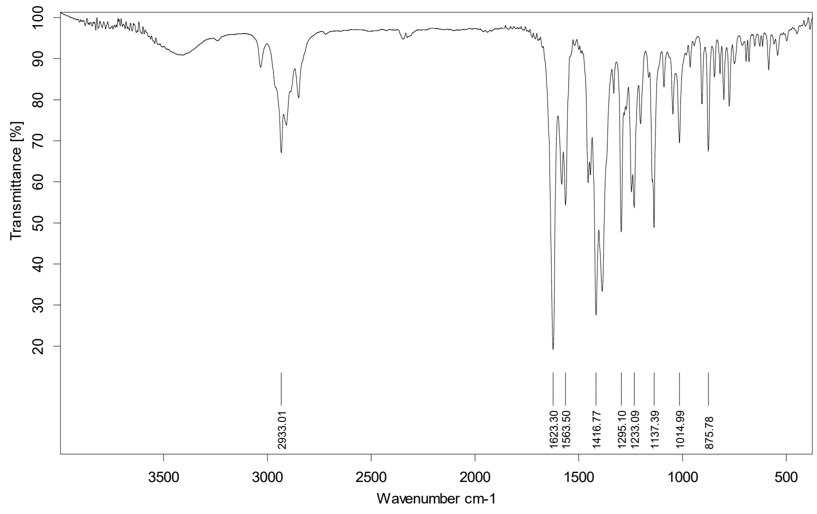


# $^1$H NMR

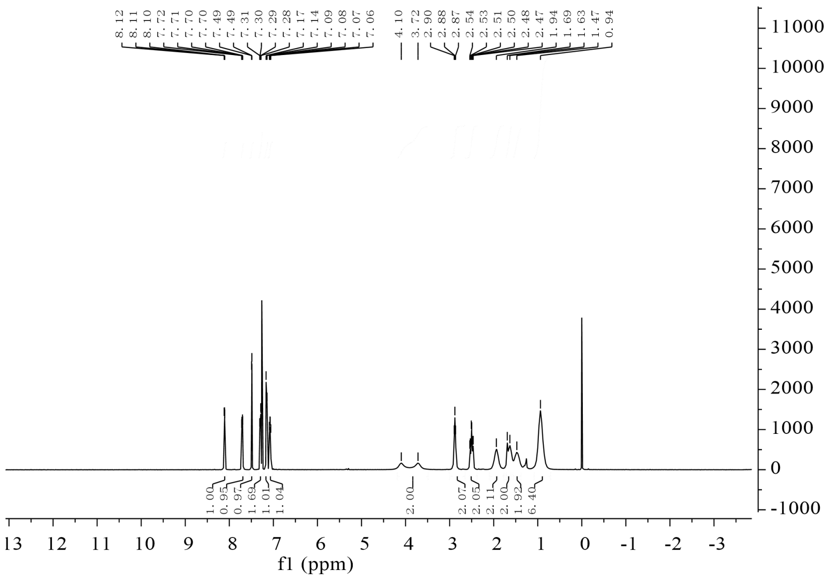

## $^{13}$C NMR

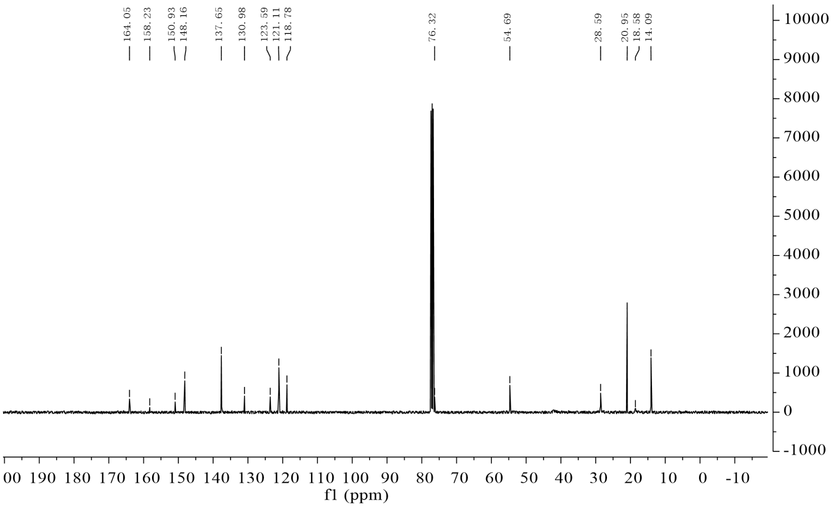


## HRMS

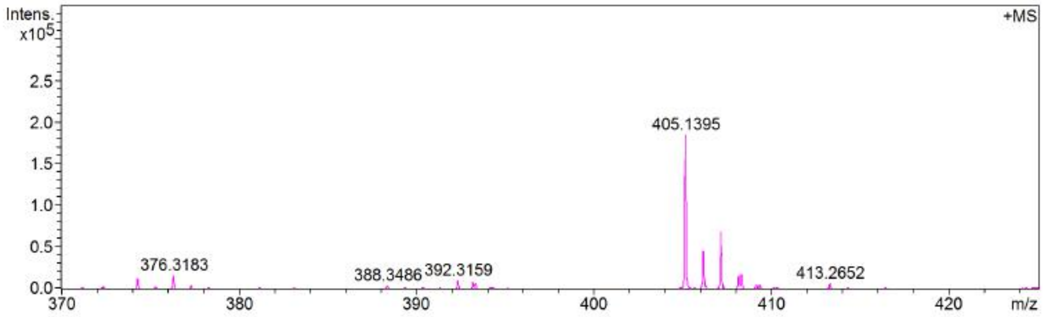


| Meas. m/z | # | Formula | m/z | err [ppm] | N-Rule |
|---|---|---|---|---|---|
| 405.1395 | 1 | C 21 H 26 Cl N 2 O 2 S | 405.1398 | 0.7 | ok |

White solid, m.p. 112-113 ℃; IR (KBr, $cm^{-1}$) *v*: 2933 (C-H), 1623 (C=O), 1563-1416 (C=C), 1233 (C-O); $^{1}$H NMR (400 MHz, $CDCl_3$) δ 8.15-8.06 (m, 1H, Py-H), 7.71 (dd, *J* = 7.3, 1.8 Hz, 1H, Py-H), 7.49 (d, *J* = 2.4 Hz, 1H, Ar-H), 7.29 (dd, *J* = 8.6, 2.5 Hz, 2H, Ar-H), 7.16 (d, *J* = 8.7 Hz, 1H, Ar-H), 7.07 (dd, *J* = 7.3, 4.9 Hz, 1H, Py-H), 3.91 (d, *J* = 152.2 Hz, 2H, N-$CH_2$), 2.87 (d, *J* = 6.1 Hz, 2H, S-$CH_2$), 2.50-1.94 (m, 4H, -$CH_2$-), 1.66-1.47 (m, 4H, -C-$CH_2$-), 0.94 (s, 6H, -C-$CH_3$); $^{13}$C NMR (101 MHz, $CDCl_3$) δ 164.05, 158.23, 150.93, 148.16, 137.65, 130.98, 123.59, 121.11, 118.78, 54.69, 28.59, 20.95, 18.58, 14.09; HRMS calcd. for [M + $H^+$] $C_{21}H_{26}ClN_2O_2S$: 405.1398, found 405.1395.

# Compound 4s

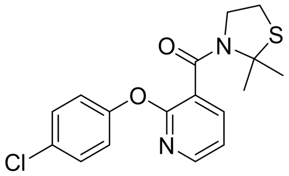


*(2-(4-chlorophenoxy)pyridin-3-yl)(2,2-dimethylthiazolidin-3-yl)methanone*

# IR

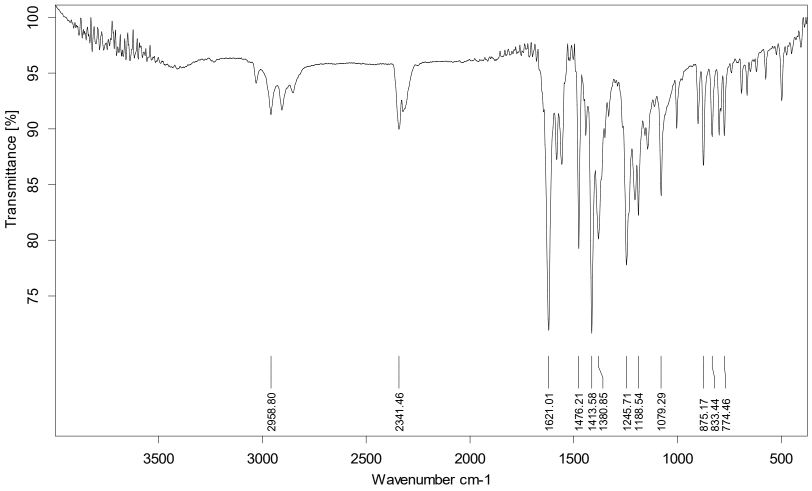


# $^{1}$H NMR

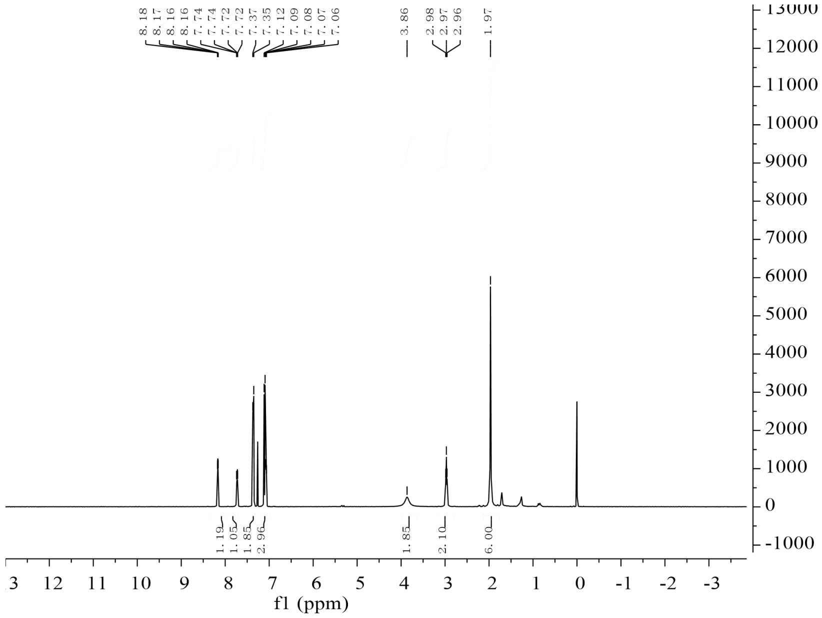

# $^{13}$C NMR

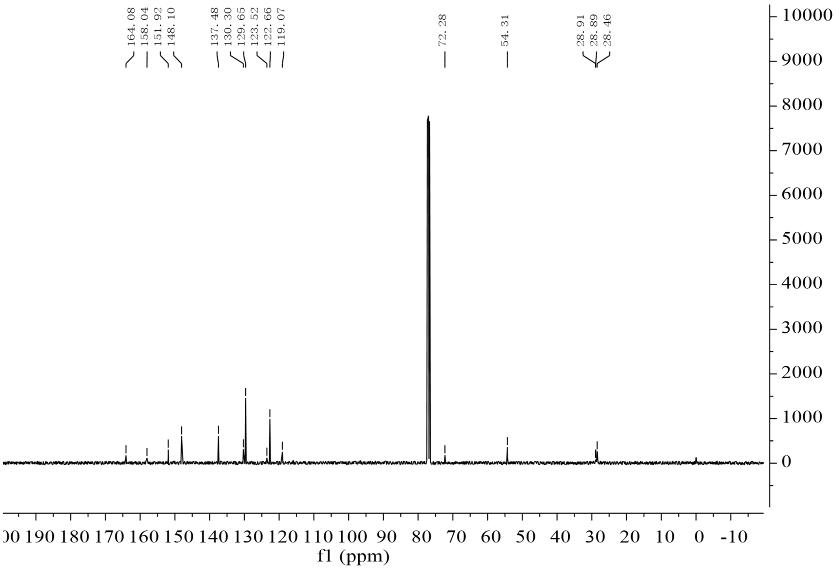


# HRMS

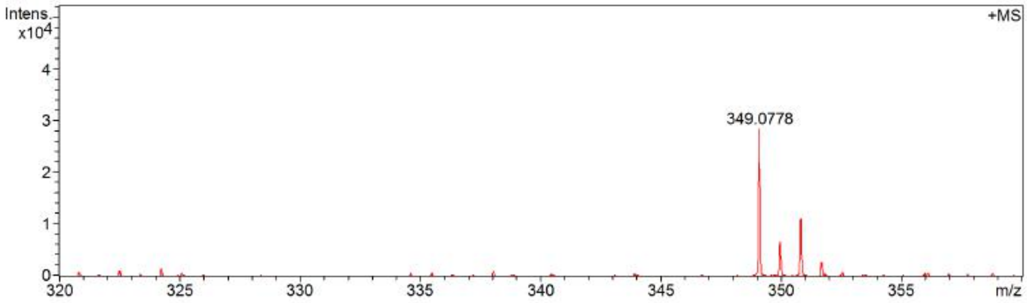


| Meas. m/z | # | Formula | m/z | err [ppm] | N-Rule |
|---|---|---|---|---|---|
| 349.0778 | 1 | C 17 H 18 Cl N 2 O 2 S | 349.0772 | -1.7 | ok |

White solid, m.p. 117-118 ℃; IR (KBr, cm$^{-1}$) $\nu$: 2958 (C-H), 1621 (C=O), 1551-1476 (C=C), 1245 (C-O); $^{1}$H NMR (400 MHz, $CDCl_3$) δ 8.17 (dd, $J$ = 5.0, 1.9 Hz, 1H, Py-H), 7.73 (dd, $J$ = 7.3, 1.9 Hz, 1H, Py-H), 7.36 (d, $J$ = 8.9 Hz, 2H, Ar-H), 7.20-7.00 (m, 3H, Ar-H, Py-H), 3.86 (s, 2H, N-$CH_2$), 2.97 (t, $J$ = 6.0 Hz, 2H,S-$CH_2$), 1.97 (s, 6H, -$(CH_3)_2$); $^{13}$C NMR (101 MHz, $CDCl_3$) δ 164.08, 158.04, 151.92, 148.10, 137.48, 130.30, 129.65, 123.52, 122.66, 119.07, 72.28, 54.31, 28.89, 28.46; HRMS calcd. for [M + H$^{+}$] $C_{17}H_{18}ClN_2O_2S$: 349.0772, found 349.0778.

# Compound 4t

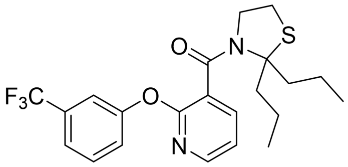


*(2-(4-chlorophenoxy)pyridin-3-yl)(2,2-diethylthiazolidin-3-yl)methanone*

# IR

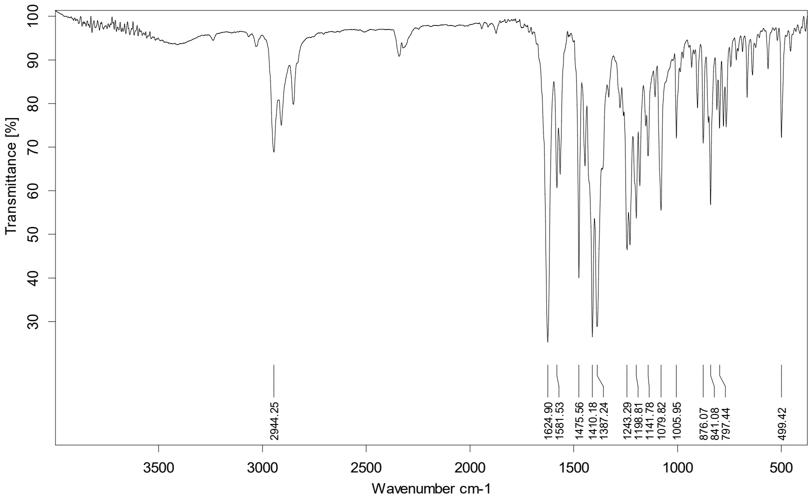


# ¹H NMR

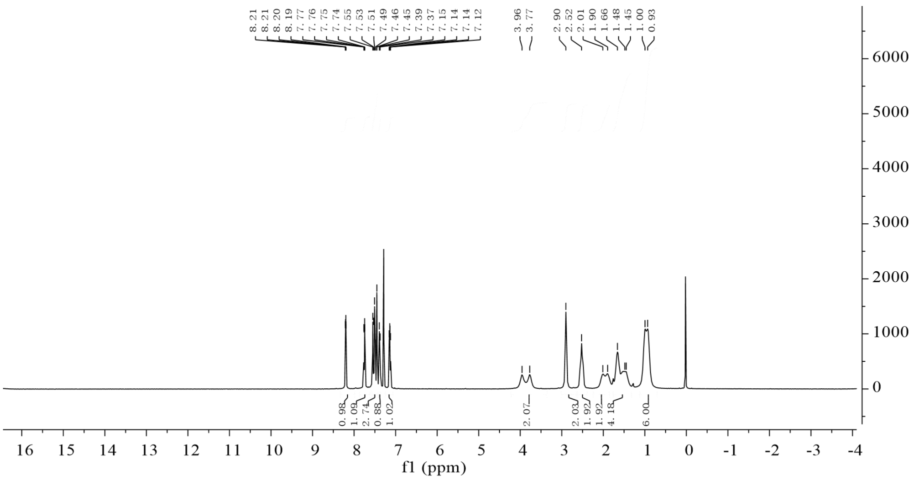

## $^{13}$C NMR

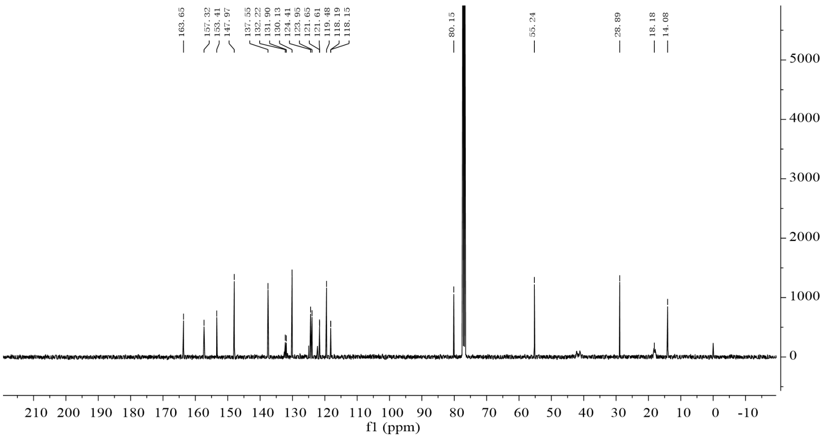


## HRMS

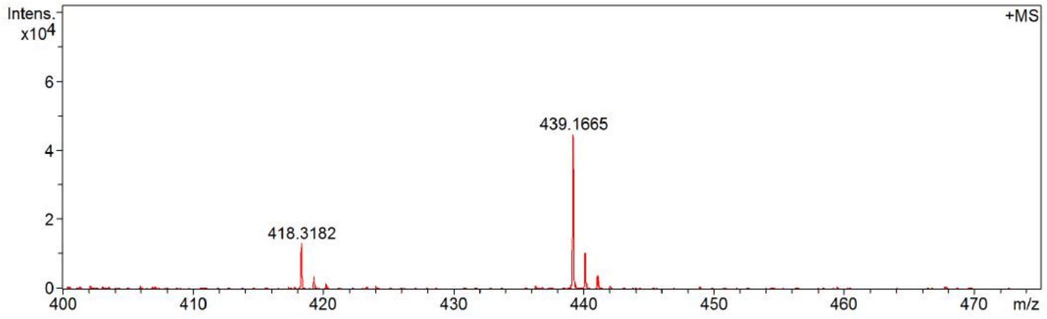


| Meas. m/z | # | Formula | m/z | err [ppm] | N-Rule |
|---|---|---|---|---|---|
| 439.1665 | 1 | C 22 H 26 F 3 N 2 O 2 S | 439.1662 | -0.8 | ok |

White solid, m.p. 91-92 ℃; IR (KBr, $cm^{-1}$) $\nu$: 2944 (C-H), 1624 (C=O), 1581-1475 (C=C), 1243 (C-O); $^{1}$H NMR (400 MHz, $CDCl_3$) δ 8.16 (dd, $J$ = 4.9, 1.9 Hz, 1H, Py-H), 7.71 (dd, $J$ = 7.3, 1.9 Hz, 1H, Py-H), 7.36 (d, $J$ = 8.8 Hz, 2H, Ar-H), 7.15-6.96 (m, 3H, Ar-H, Py-H), 3.86 (d, $J$ = 84.5 Hz, 2H, N-$CH_2$), 2.87 (d, $J$ = 6.2 Hz, 2H, S-$CH_2$), 2.61-1.97 (m, 4H, -$CH_2$-), 1.08 (t, $J$ = 7.3 Hz, 6H, -C-$CH_3$); $^{13}$C NMR (101 MHz, $CDCl_3$) δ 163.88, 157.77, 151.67, 147.98, 137.43, 130.33, 123.71, 122.64, 119.07, 81.51, 55.43, 28.83; HRMS calcd. for [M + $H^+$] $C_{22}H_{26}F_3N_2O_2S$: 439.1662, found 439.1665.

# Compound 4u

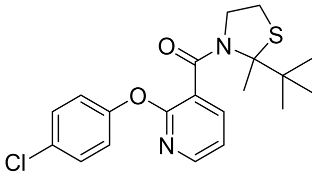

*(2-(tert-butyl)-2-methylthiazolidin-3-yl)(2-(4-chlorophenoxy)pyridin-3-yl)methanone*

# IR

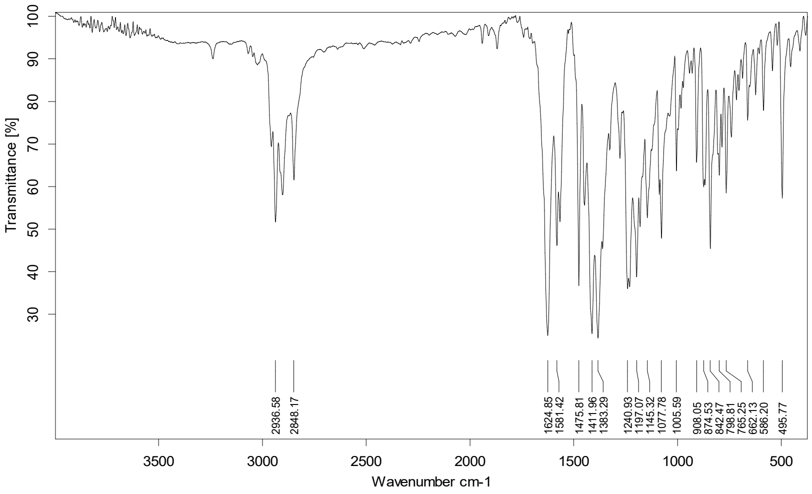

# $^1$H NMR

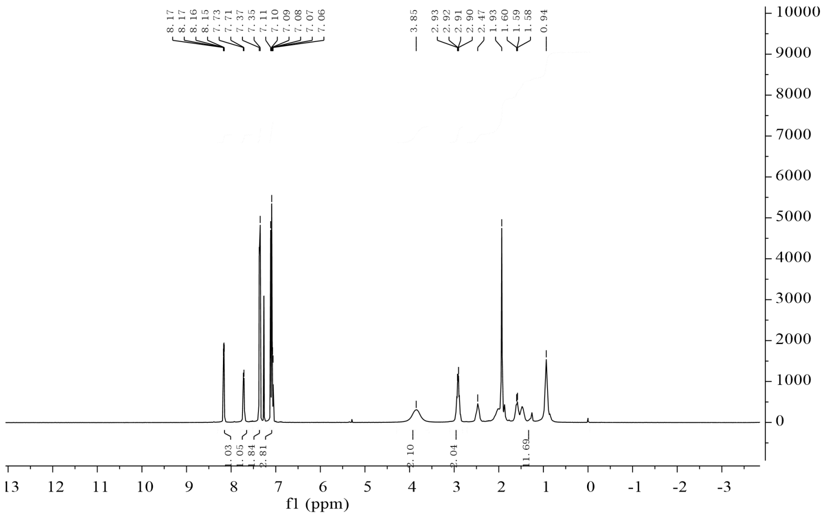

## $^{13}$C NMR

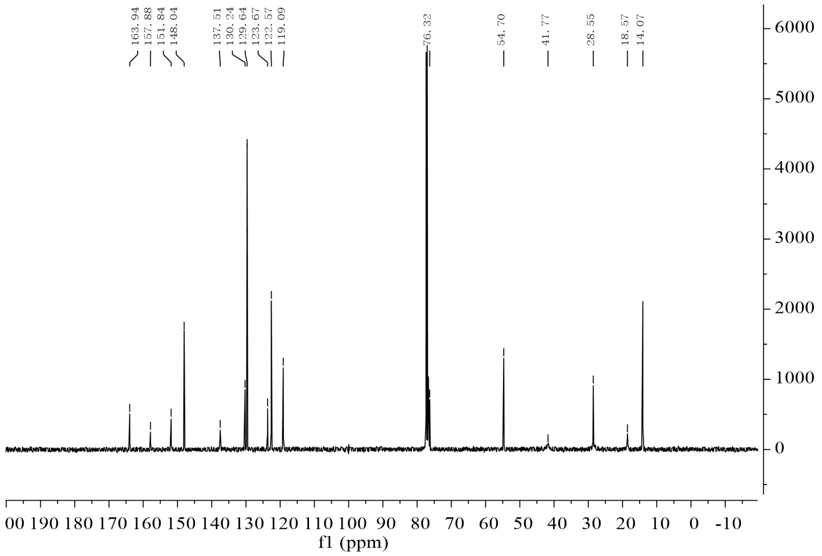

## HRMS

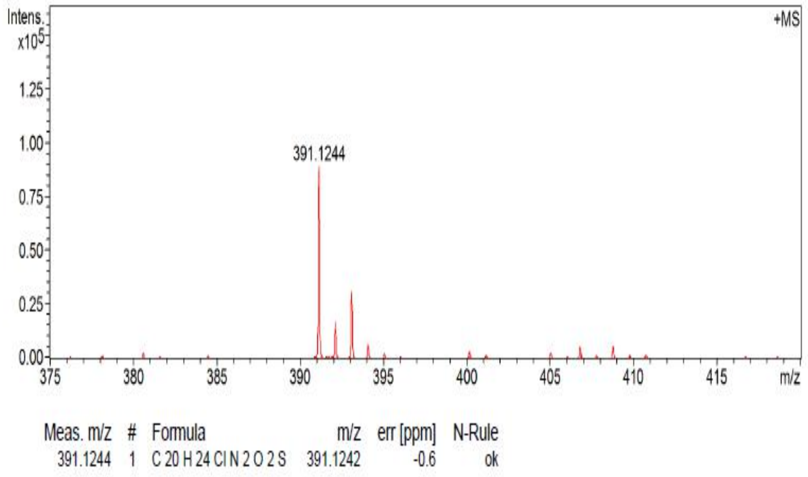


White solid, m.p. 86-88 ℃; IR (KBr, $cm^{-1}$) $\nu$: 2936-2848 (C-H), 1624 (C=O), 1581-1475 (C=C), 1240 (C-O); $^1$H NMR (400 MHz, $CDCl_3$) δ 8.16 (dd, $J$ = 4.9, 1.9 Hz, 1H, Py-H), 7.72 (d, $J$ = 5.5 Hz, 1H, Py-H), 7.36 (d, $J$ = 8.8 Hz, 2H, Ar-H), 7.13 – 7.05 (m, 3H, Ar-H, Py-H), 3.85 (s, 2H, N-$CH_2$), 3.11-2.81 (m, 2H, S-$CH_2$), 2.47-0.66 (m, 12H, -$CH_3$, -$(CH_3)_3$); $^{13}$C NMR (101 MHz, $CDCl_3$) δ 163.94, 157.88, 151.84, 148.04, 137.51, 130.24, 129.64, 123.67, 122.57, 119.09, 54.70, 28.55, 18.57, 14.07; HRMS calcd. for [M + $H^+$] $C_{20}H_{24}ClN_2O_2S$: 391.1242, found 391.1244.

# Compound 4v

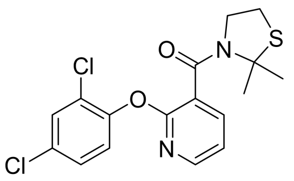


*(2-(2,4-dichlorophenoxy)pyridin-3-yl)(2,2-dimethylthiazolidin-3-yl)methanone*

# IR

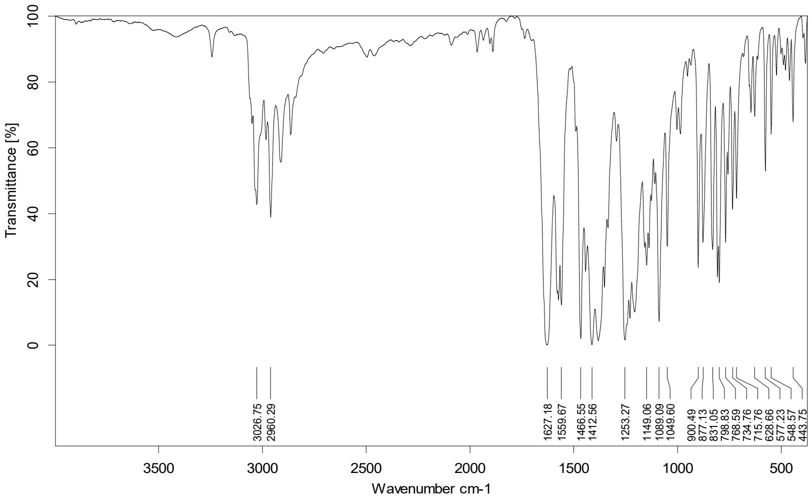


# $^1$H NMR

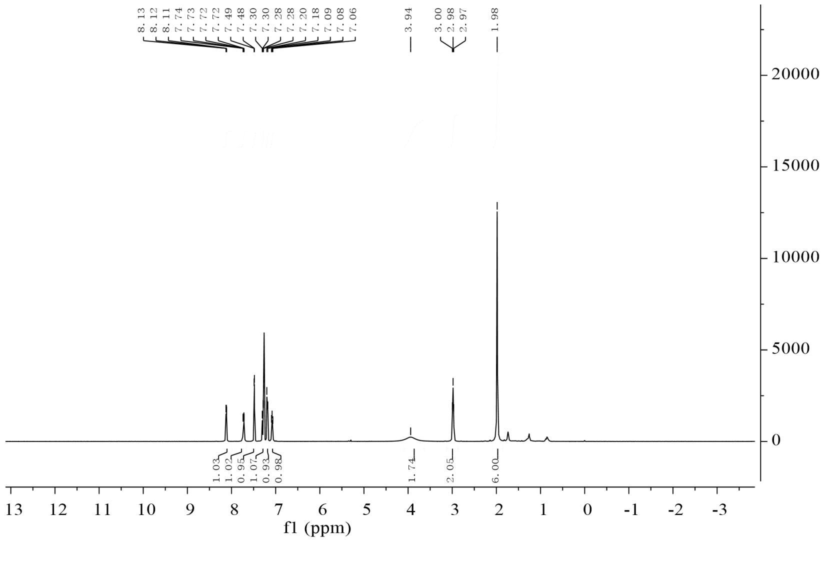

## $^{13}C$ NMR

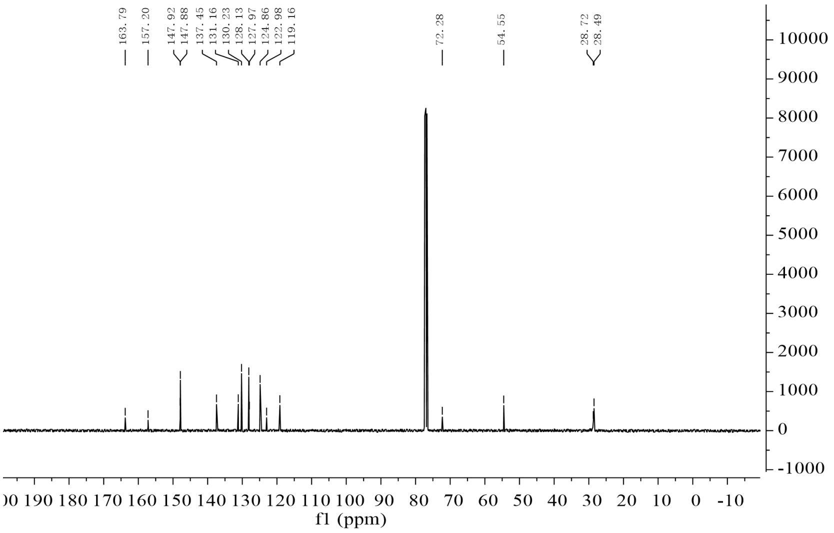


## HRMS

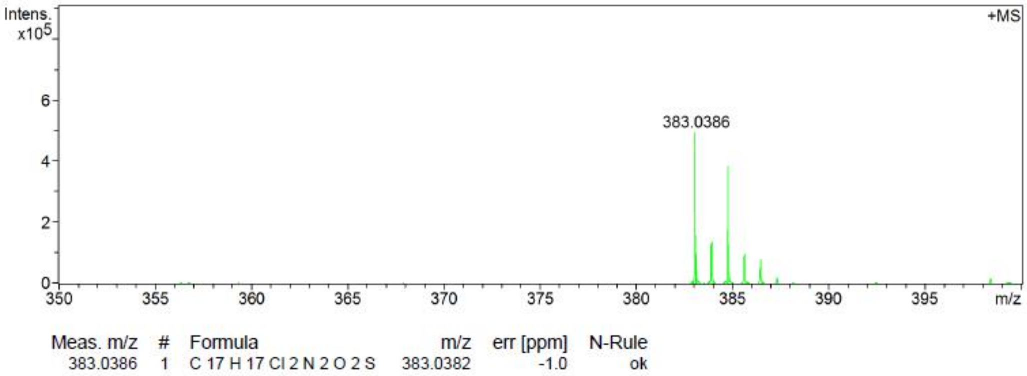


White solid, m.p. 121-122 ℃； IR (KBr, $cm^{-1}$) $\nu$: 3029 -2966(C-H), 1627 (C=O), 1559-1466 (C=C), 1253 (C-O); $^{1}H$ NMR (400 MHz, $CDCl_3$) δ 8.22-7.99 (m, 1H, Py-H), 7.89 – 7.66 (m, 1H, Py-H), 7.48 (d, $J$ = 2.4 Hz, 1H, Ar-H), 7.29 (dd, $J$ = 8.7, 2.4 Hz, 1H, Ar-H), 7.19 (d, $J$ = 8.7 Hz, 1H, Ar-H), 7.12 – 7.03 (m, 1H, Py-H), 3.94 (s, 2H, N-$CH_2$), 2.98 (t, $J$ = 5.9 Hz, 2H S-$CH_2$), 1.98 (s, 6H, -$(CH_3)_2$); $^{13}C$ NMR (101 MHz, $CDCl_3$) δ 163.79, 157.20, 147.92, 147.88, 137.45, 131.16, 130.23, 128.13, 127.97, 124.86, 122.98, 119.16, 72.28, 54.55, 28.72, 28.49; HRMS calcd. for [M + $H^+$] $C_{17}H_{17}Cl_2N_2O_2S$: 383.0382, found 383.0386.

# Compound 4w

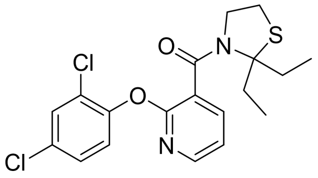


*(2-(2,4-dichlorophenoxy)pyridin-3-yl)(2,2-diethylthiazolidin-3-yl)methanon*

# IR

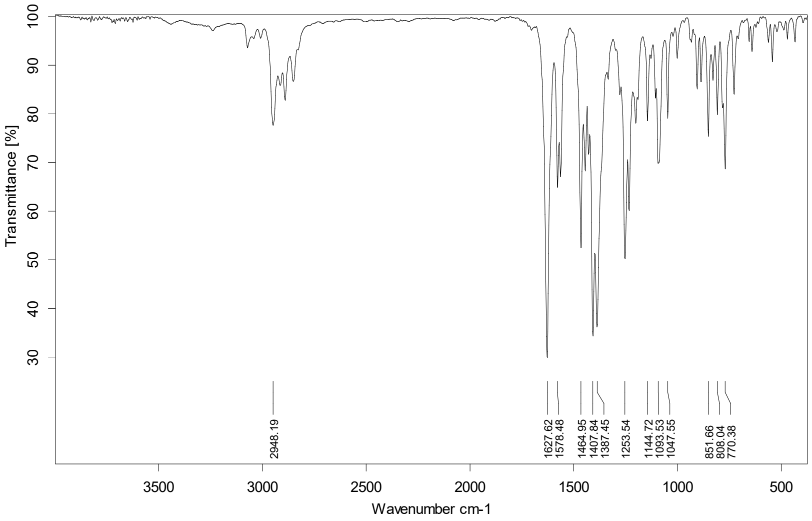


# $^1$H NMR

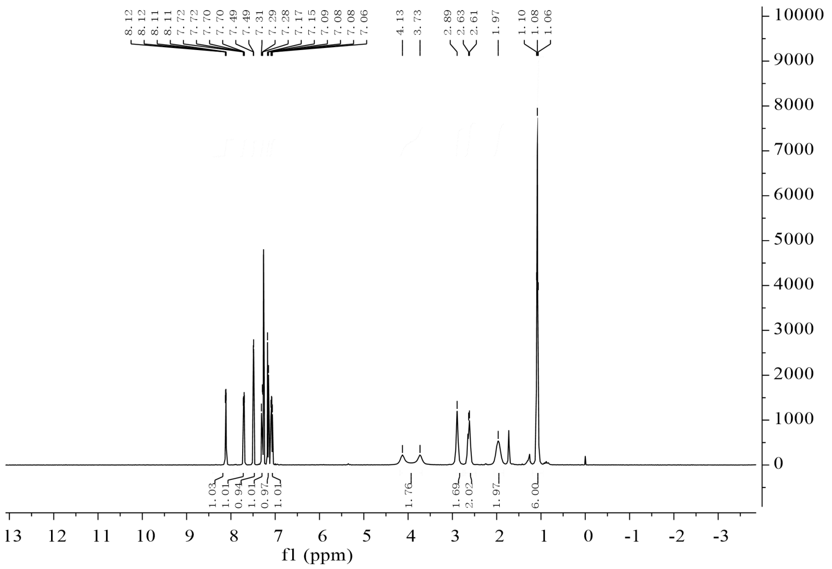

# $^{13}$C NMR

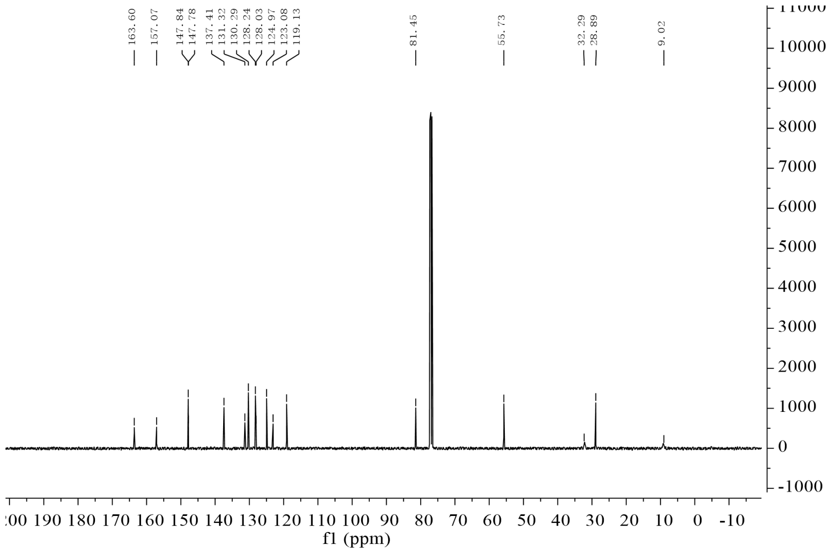


# HRMS

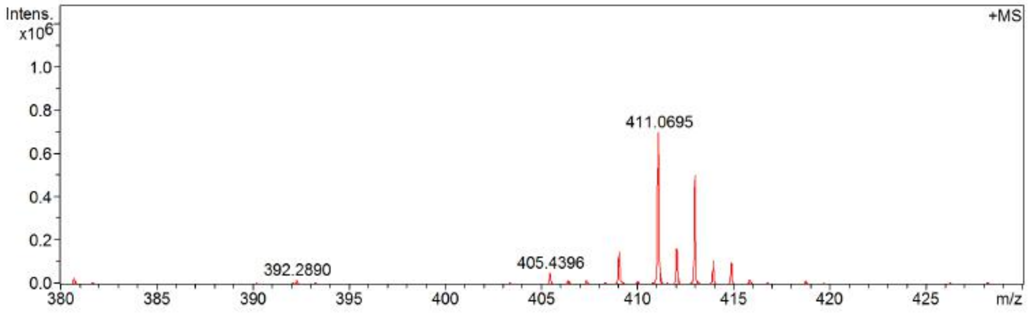


| Meas. m/z | # | Formula | m/z | err [ppm] | N-Rule |
|---|---|---|---|---|---|
| 411.0695 | 1 | C 19 H 21 Cl 2 N 2 O 2 S | 411.0695 | 0.1 | ok |

White solid, m.p. 75-77 ℃; IR (KBr, $cm^{-1}$) $\nu$: 2948 (C-H), 1627 (C=O), 1578-1464 (C=C), 1253 (C-O); $^{1}$H NMR (400 MHz, $CDCl_3$) δ 8.12 (dd, $J$ = 5.1, 1.8 Hz, 1H, Py-H), 7.71 (dd, $J$ = 7.3, 1.8 Hz, 1H, Py-H), 7.49 (d, $J$ = 2.4 Hz, 1H, Ar-H), 7.33-7.27 (m, 1H, Ar-H), 7.16 (d, $J$ = 8.6 Hz, 1H, Ar-H), 7.08 (dd, $J$ = 7.3, 4.9 Hz, 1H, Py-H), 3.93 (d, $J$ = 158.6 Hz, 2H, N-$CH_2$), 2.89 (s, 2H, S-$CH_2$), 2.62-1.97 (m, 4H, -$CH_2$-), 1.08 (t, $J$ = 7.2 Hz, 6H,-C-$CH_3$); $^{13}$C NMR (101 MHz, $CDCl_3$) δ 163.60, 157.07, 147.84, 147.78, 137.41, 131.32, 130.29, 128.24, 128.03, 124.97, 123.08, 119.13, 81.45, 55.73, 32.29, 28.89; HRMS calcd. for [M + $H^+$] $C_{19}H_{21}Cl_2N_2O_2S$: 411.0695, found 411.0695.

# Compound 4x

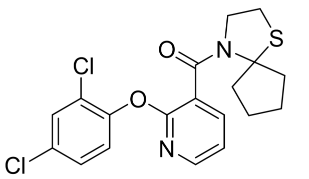


*(2-(2,4-dichlorophenoxy)pyridin-3-yl)(1-thia-4-azaspiro[4.4]nonan-4-yl)methanone*

# IR

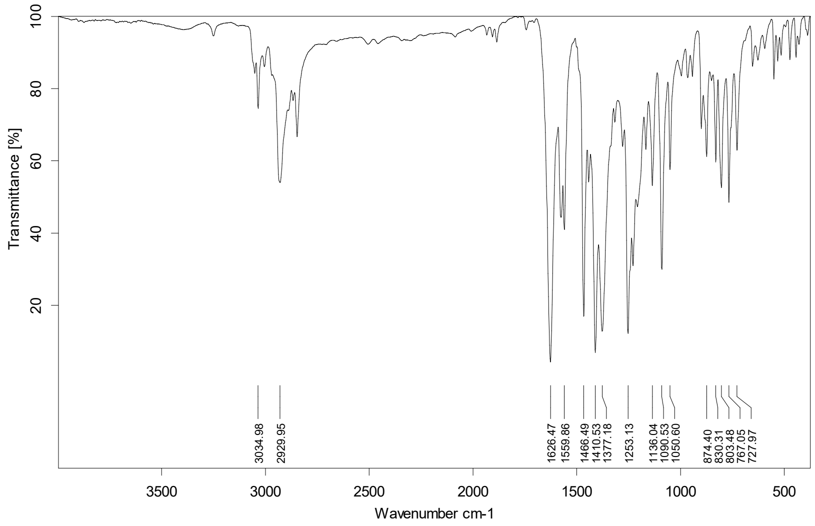


# $^1$H NMR

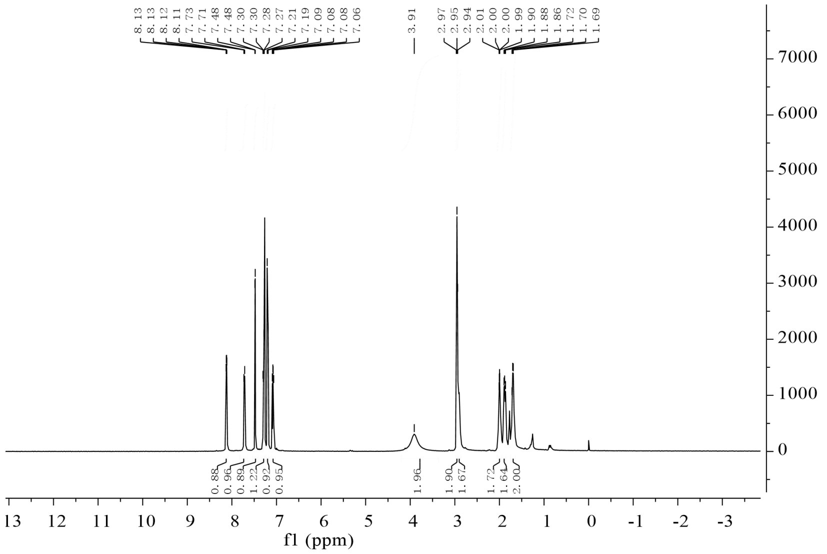

## $^{13}$C NMR

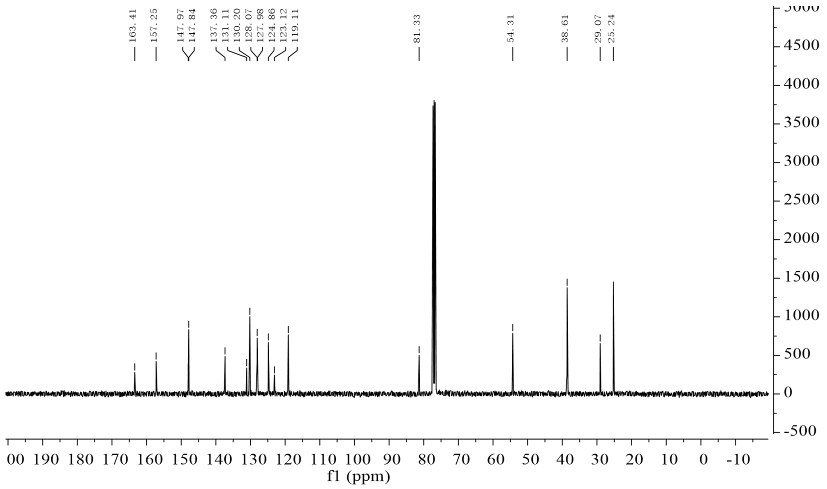


## HRMS

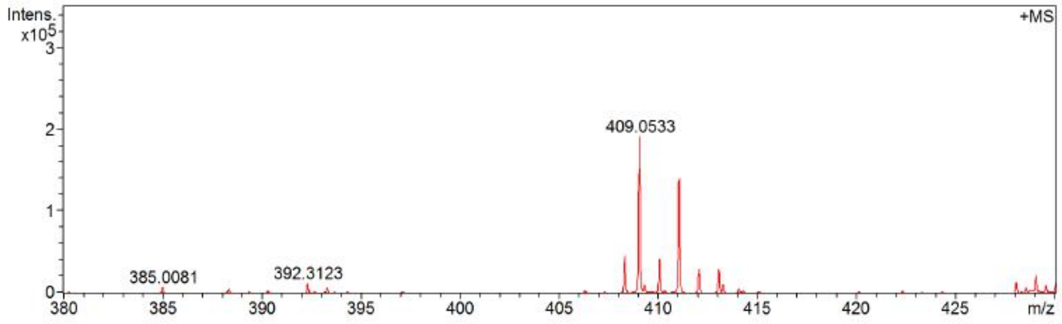


| Meas. m/z | # | Formula | m/z | err [ppm] | N-Rule |
|---|---|---|---|---|---|
| 409.0533 | 1 | C 19 H 19 Cl 2 N 2 O 2 S | 409.0539 | 1.4 | ok |

White solid, m.p. 95-97 ℃; IR (KBr, cm$^{-1}$) $\nu$: 3034-2929 (C-H), 1626 (C=O), 1556-1466 (C=C), 1253 (C-O); $^{1}$H NMR (400 MHz, $CDCl_3$) δ 8.12 (dd, $J$ = 4.9, 1.7 Hz, 1H, Py-H), 7.72 (d, $J$ = 7.3 Hz, 1H, Py-H), 7.48 (d, $J$ = 2.4 Hz, 1H, Ar-H), 7.29 (dd, $J$ = 8.7, 2.4 Hz, 1H, Ar-H), 7.20 (d, $J$ = 8.7 Hz, 1H, Ar-H), 7.08 (dd, $J$ = 7.3, 5.0 Hz, 1H, Py-H), 3.91 (s, 2H, N-$CH_2$), 2.95 (t, $J$ = 6.0 Hz, 2H, S-$CH_2$), 2.90-1.95 (m, 4H, -$CH_2$-), 1.87-1.63 (m, 4H, -$CH_2$-); $^{13}$C NMR (101 MHz, $CDCl_3$) δ 163.41, 157.25, 147.97, 147.84, 137.36, 131.11, 130.20, 128.07, 127.98, 124.86, 119.11, 81.33, 54.31, 38.61, 29.07, 25.24; HRMS calcd. for [M + H$^+$] $C_{19}H_{19}Cl_2N_2O_2S$: 409.0539, found 409.0533.

# Compound 4y

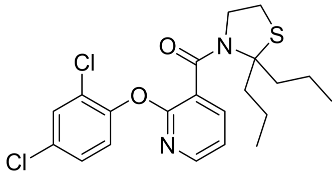

*2-(2,4-dichlorophenoxy)pyridin-3-yl)(2,2-dipropylthiazolidin-3-yl)methanone*

# IR

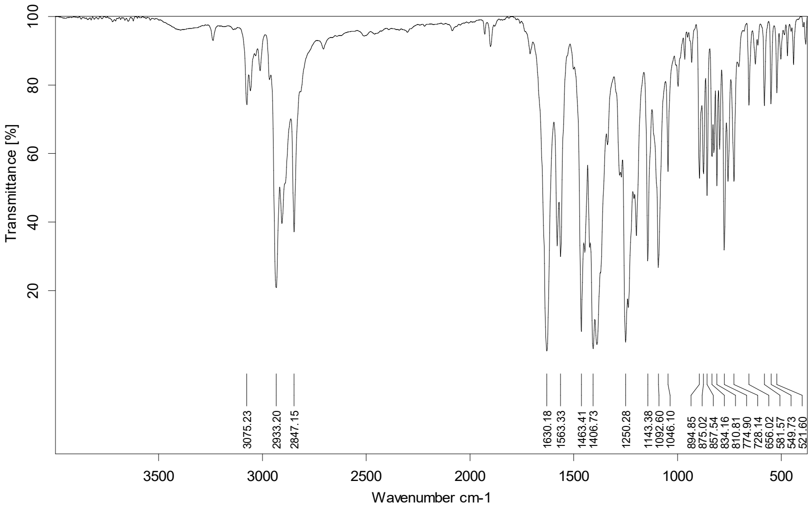

# $^1$H NMR

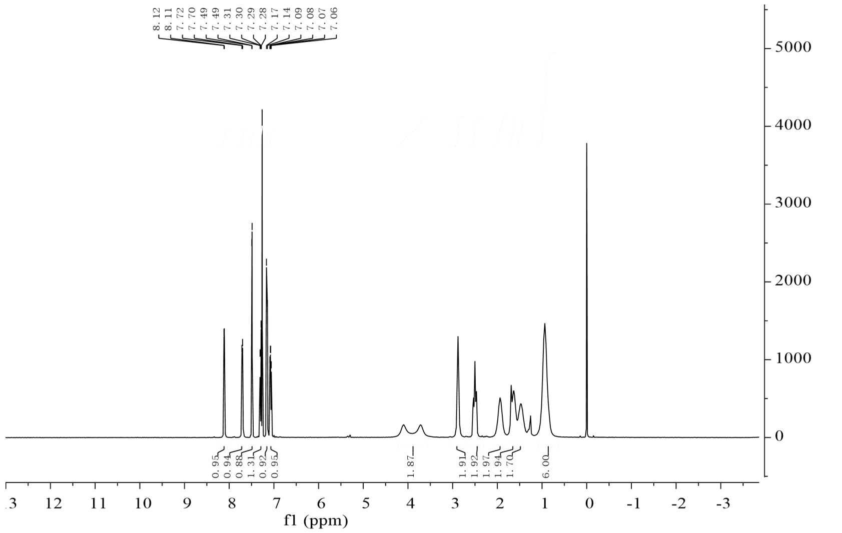

## $^{13}$C NMR

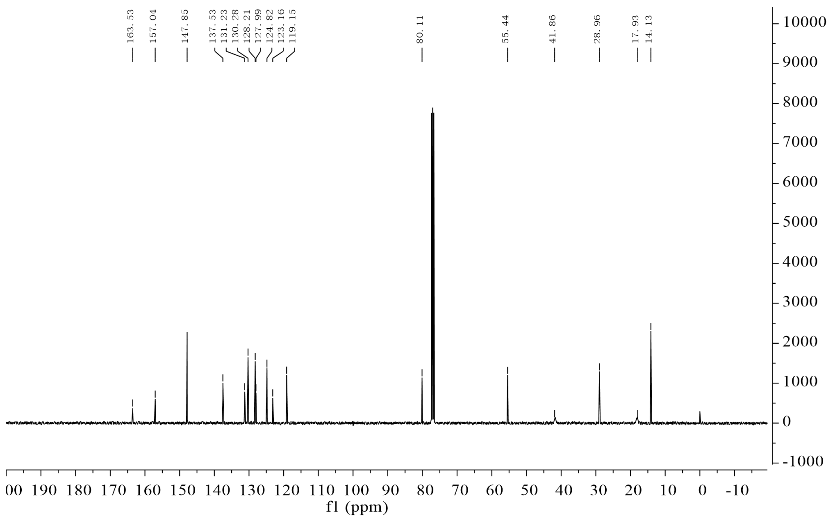


## HRMS

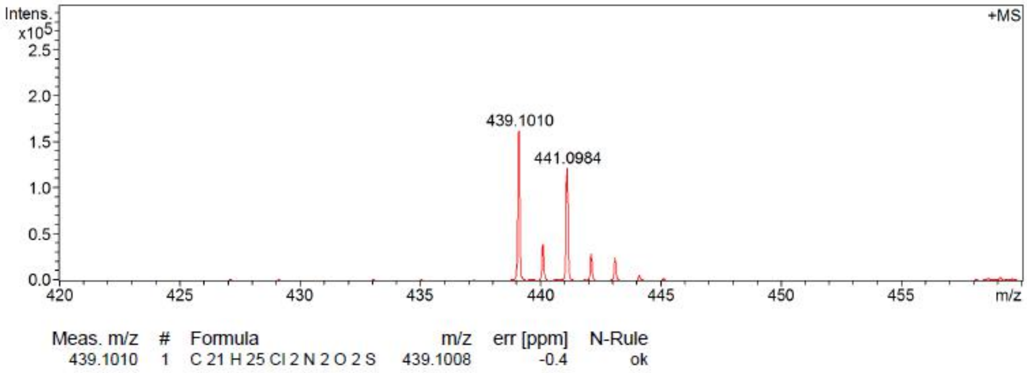


White solid, m.p. 112-114 ℃; IR (KBr, cm$^{-1}$) *v*: 3075-2847 (C-H), 1630 (C=O), 1563-1463 (C=C), 1250 (C-O); $^{1}$H NMR (400 MHz, $CDCl_3$) δ 8.11 (d, *J* = 3.3 Hz, 1H, Py-H), 7.71 (d, *J* = 6.2 Hz, 1H, Py-H), 7.49 (d, *J* = 2.4 Hz, 1H, Ar-H), 7.29 (dd, *J* = 8.7, 2.4 Hz, 1H, Ar-H), 7.16 (d, *J* = 8.7 Hz, 1H, Ar-H), 7.07 (dd, *J* = 7.1, 5.0 Hz, 1H, Py-H), 3.91 (d, *J* = 152.4 Hz, 2H, N-$CH_2$), 2.88 (t, *J* = 6.0 Hz, 2H, S-$CH_2$), 2.50-1.94 (m, 4H, -$CH_2$-), 1.66-1.47 (s, 4H, -C-$CH_2$-), 0.94 (s, 6H, -C-$CH_3$); $^{13}$C NMR (101 MHz, $CDCl_3$) δ 163.53, 157.04, 147.85, 137.53, 131.23, 130.28, 128.21, 127.99, 124.82, 123.16, 119.15, 80.11, 55.44, 28.96, 14.13; HRMS calcd. for [M + H$^+$] $C_{21}H_{25}Cl_2N_2O_2S$: 439.1008, found 439.1010.

# Compound 4z

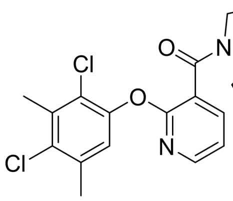

*(2-(4-chloro-3,5-dimethylphenoxy)pyridin-3-yl)(1-thia-4-azaspiro[4.4]nonan-4-yl)methanone*

# IR

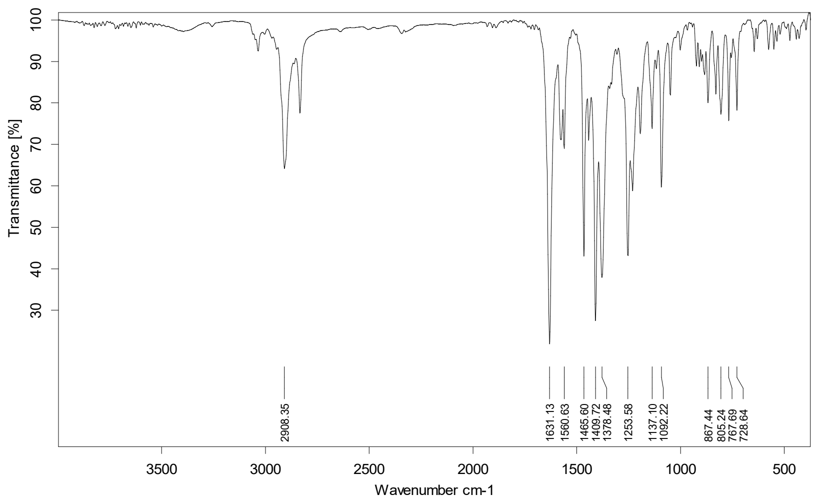

# $^{1}$H NMR

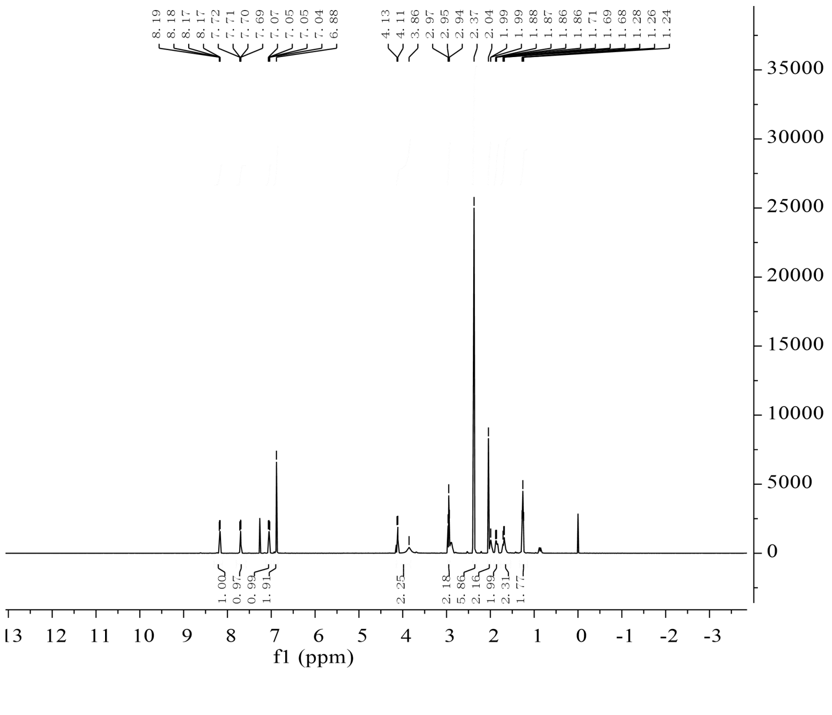

## $^{13}$C NMR

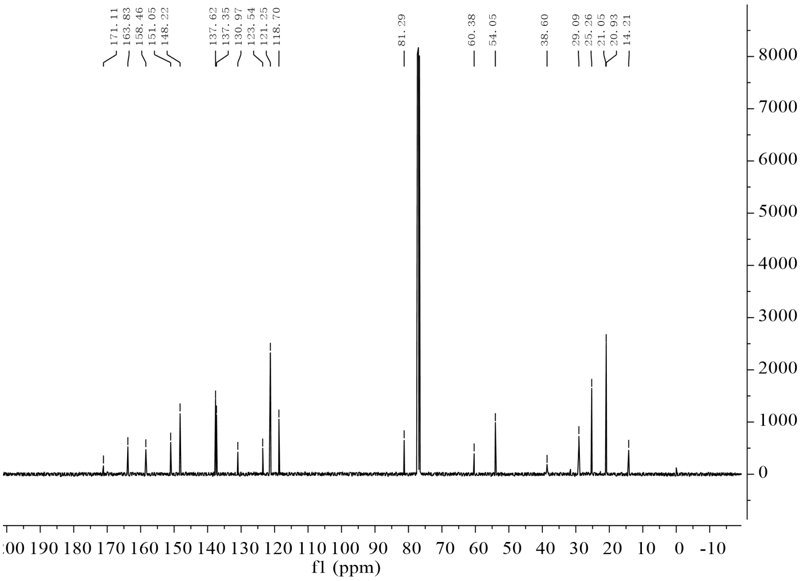


## HRMS

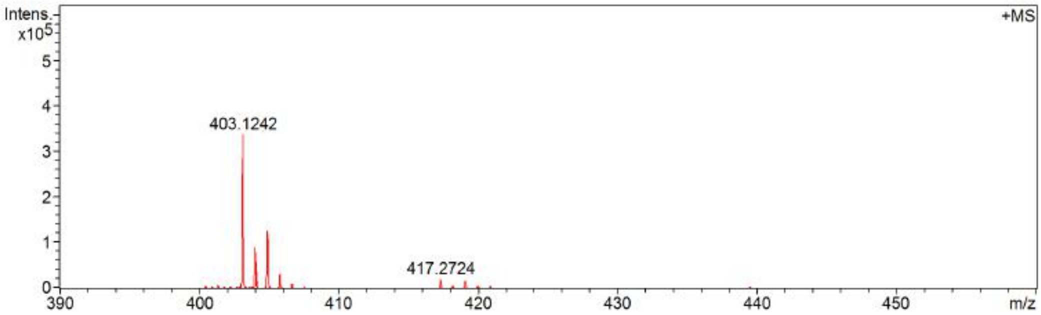


| Meas. m/z | # | Formula | m/z | err [ppm] | N-Rule |
|---|---|---|---|---|---|
| 403.1242 | 1 | C 21 H 24 Cl N 2 O 2 S | 403.1242 | -0.1 | ok |

White solid, m.p. 117-119 ℃; IR (KBr, cm$^{-1}$) $\nu$: 2908 (C-H), 1631 (C=O), 1566-1465 (C=C), 1253 (C-O); $^{1}$H NMR (400 MHz, $CDCl_3$ ) δ 8.18 (dd, $J$ = 4.9, 1.9 Hz, 1H, Py-H), 7.71 (dd, $J$ = 7.3, 2.0 Hz, 1H, Py-H), 7.05 (dd, $J$ = 7.3, 5.0 Hz, 1H, Ar-H), 6.88 (s, 2H, Ar-H, Py-H), 4.21-3.84 (m, 2H, N-$CH_2$), 2.95 (t, $J$ = 6.0 Hz, 2H, S-$CH_2$), 2.37-1.26 (m, 12 H, -$CH_3$, -$(CH_3)_3$); $^{13}$C NMR (101 MHz, $CDCl_3$) δ 163.83, 151.05, 148.22, 137.62, 137.35, 123.54, 121.25, 118.70, 81.29, 60.38, 54.05, 38.60, 29.09, 25.26, 20.93, 14.21; HRMS calcd. for [M + H$^{+}$] $C_{21}H_{24}ClN_2O_2S$: 403.1242, found 403.1242.
